# Overview of advances in anode electrode engineering for high-energy-density lithium–sulfur batteries

**DOI:** 10.1039/d6ra06978a

**Published:** 2026-09-25

**Authors:** Anjana A., Manu U. M. Patel

**Affiliations:** a Manipal Institute of Technology, Manipal Academy of Higher Education Manipal India Manu.patel@manipal.edu

## Abstract

Lithium–sulfur batteries are regarded as promising candidates for future energy storage owing to their high theoretical energy density and the abundance of elemental sulfur. Existing research has focused on optimizing lithium–sulfur battery cathodes, electrolytes, and other components, while the role of the anode remains equally critical in determining the overall cell performance, safety, and longevity. This review provides a comprehensive overview of strategies for addressing the challenges of lithium–sulfur battery anodes. It covers key approaches, such as artificial solid electrolyte interphase layers, electrolyte engineering, lithiophilic host design, current collector modification, and the development of solid-state electrolytes. Additionally, it offers a brief overview of digital modeling and intelligent control methods. This review discusses alternative anode materials, such as silicon anodes, silicon–graphite composites, and carbon-based systems, with an emphasis on their synergistic effects in improving stability and electrochemical performance. Recent advances in anode-free configurations are also highlighted, providing insights into pathways for achieving higher energy densities alongside enhanced safety. This review further evaluates the advantages and limitations of the current strategies and outlines future directions, focusing on scalable material design, robust interface engineering, and practical implementation. Overall, this work emphasizes the critical role of anode engineering in unlocking the full potential of lithium–sulfur batteries and provides insights for the rational design of high-performance and durable energy storage systems.

## Abstract 2

Li–S battery anodes: challenges and future directions. This review critically evaluates challenges in Li-metal anodes, mitigation strategies, alternative anode materials, and emerging anode-free Li–S batteries.

## Graphical abstract

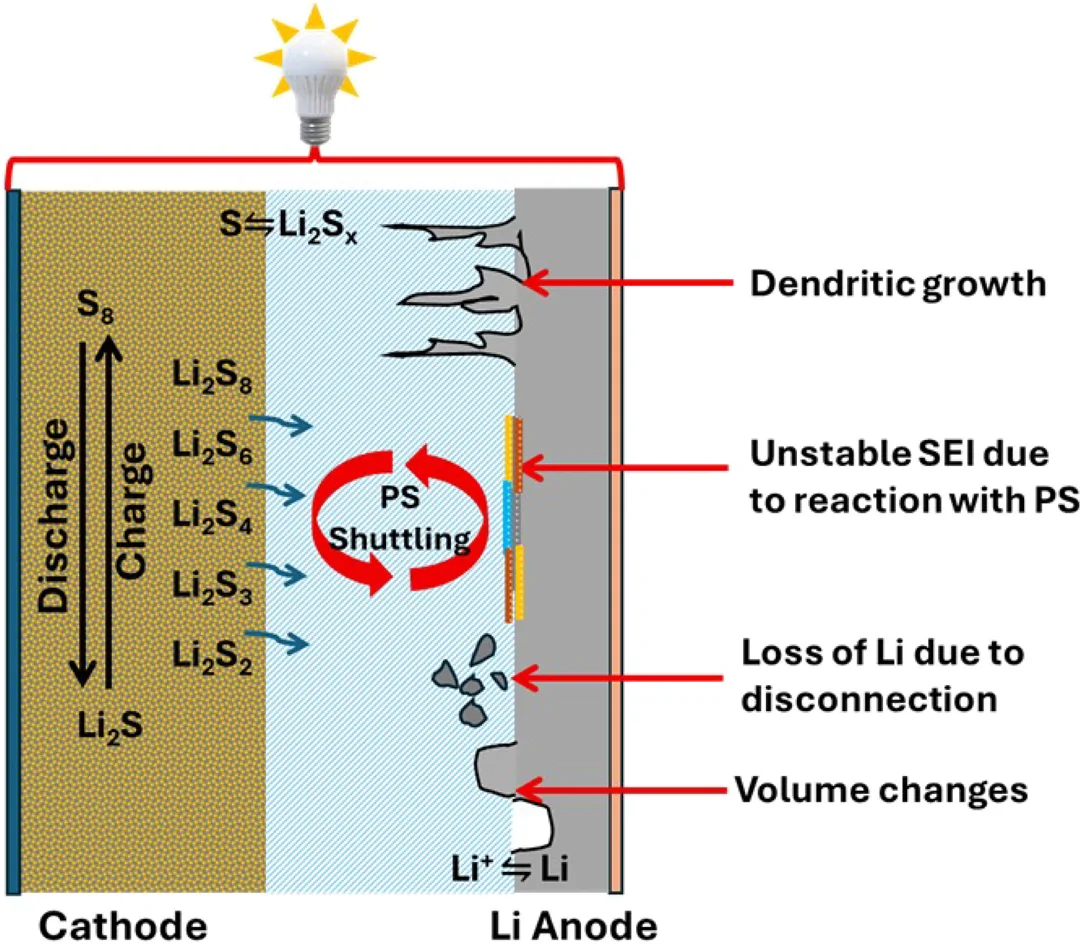

## 1. Introduction

Lithium–sulfur (Li–S) batteries are widely recognized as promising next-generation high-energy density candidates because of their high theoretical energy density of approximately 2600 Wh kg^−1^ and the large theoretical specific capacity of sulfur (1675 mA h g^−1^). These intrinsic advantages position Li–S systems as attractive alternatives to conventional battery chemistries for high-energy applications. In addition to its high energy density, sulfur (S) is a naturally abundant, low-cost, and environmentally friendly material. All these aspects make Li–S batteries a promising alternative to conventional lithium-ion (Li-ion) batteries for applications such as electric vehicles and large-scale energy storage.^1–4^ Despite these advantages, the practical implementation of Li–S batteries remains challenging due to several unaddressed challenges associated with the components (anode, cathode, electrolyte and separator) of Li–S batteries.^1,3^ Extensive research has focused on improving S cathodes by using conductive carbons and other materials to improve the conductivity, suppress polysulfide dissolution and mitigate polysulfide shuttling.^1–4^ Electrolyte design has been extensively explored to enhance ionic conductivity, suppress polysulfide shuttling and stabilize the lithium anode.^5^ Similarly, separator engineering, particularly the development of ion-selective membranes has been investigated to effectively block polysulfide migration and improve overall cell performance.^6^ Despite these advances, the pivotal role of the anode in governing the stability, safety, and electrochemical behavior of Li–S batteries has only recently received significant attention.^7–15^

In conventional Li–S battery configurations, metallic lithium (Li) is commonly used as the anode due to its extremely high theoretical capacity (3860 mA h g^−1^) and the low electrochemical potential among known anode materials.^7^ However, the use of Li introduces several critical challenges that limit the long-term performance and practical deployment of Li–S batteries. These include uncontrolled dendritic Li growth during repeated cycling, instability of the solid electrolyte interphase (SEI), and parasitic reactions between Li and dissolved polysulfide (PS) intermediates. In addition, substantial volume fluctuations during cycling lead to pronounced morphological changes, further compromising the interfacial stability and durability of the cell. Such issues lead to continuous Li consumption, low coulombic efficiency, rapid capacity fading and serious safety concerns.^8–15^ Use of metallic Li raises significant safety concerns due to its extreme reactive nature with oxygen in presence of organic solvents leading to fire accidents.^7^

To overcome these limitations, extensive research has focused on developing effective strategies to stabilize Li–S battery anodes and enhance their electrochemical reversibility and cycling durability. This review provides a comprehensive overview of the recent advances in the design and development of anodes for Li–S batteries. This review begins by outlining the fundamental challenges associated with Li anodes in Li–S batteries, including dendrite formation, interfacial instability, and PS-induced side reactions. It then provides a comprehensive overview of the key strategies developed to enhance the cyclability of metallic Li and improve interfacial stability, such as artificial SEI formation, electrolyte engineering, and host-structured anodes. We briefly discuss how digital modeling and intelligent control methods have evolved in shaping optimized Li anode electrodes. In addition, recent progress in alternative anode materials, such as silicon, silicon–graphite composites, and carbon-based systems, is summarized with an emphasis on their potential to deliver high energy densities with improved safety. Emerging anode-free Li–S configurations are also discussed, highlighting both their advantages and the critical challenges that remain to be addressed. This review further evaluates the benefits and limitations of these approaches for achieving stable Li–S anodes. Finally, it outlines future perspectives and research directions focused on scalable material design, robust interface engineering, and the realization of safe, durable, and high-energy-density Li–S battery systems.

## 2. Working principle and challenges in Li–S battery anodes

Li–S battery anode is composed of metallic Li functioning as both the active material and Li-ion reservoir by undergoing reversible plating and stripping during battery cycling. During discharge, Li is oxidized at the anode to generate Li^+^ ions and electrons. The Li^+^ ions migrate through the electrolyte to the S cathode, where elemental S is progressively reduced through the formation of a series of soluble PS intermediates (Li_2_S_8_ to Li_2_S_2_) that ultimately form insoluble Li_2_S, as described in eqn (1)–(6). Upon charging, these reactions are reversed, regenerating S at the cathode and depositing Li back onto the anode, Fig. 1.^4,5,16^

1 S_8_ + 2Li^+^ + 2e^−^ → Li_2_S_8_

2 Li_2_S_8_ + 2/3Li^+^ + 2/3e^−^ → 4/3Li_2_S_6_

3 4/3Li_2_S_6_ + 4/3Li^+^ + 4/3e^−^ → 2Li_2_S_4_

4 2Li_2_S_4_ + 4/3Li^+^ + 4/3e^−^ → 8/3Li_2_S_3_

5 8/3Li_2_S_3_ + 8/3Li^+^ + 8/3e^−^ → 4Li_2_S_2_

6 4Li_2_S_2_ + 8Li^+^ + 8e^−^ → 8Li_2_S

**Fig. 1:**
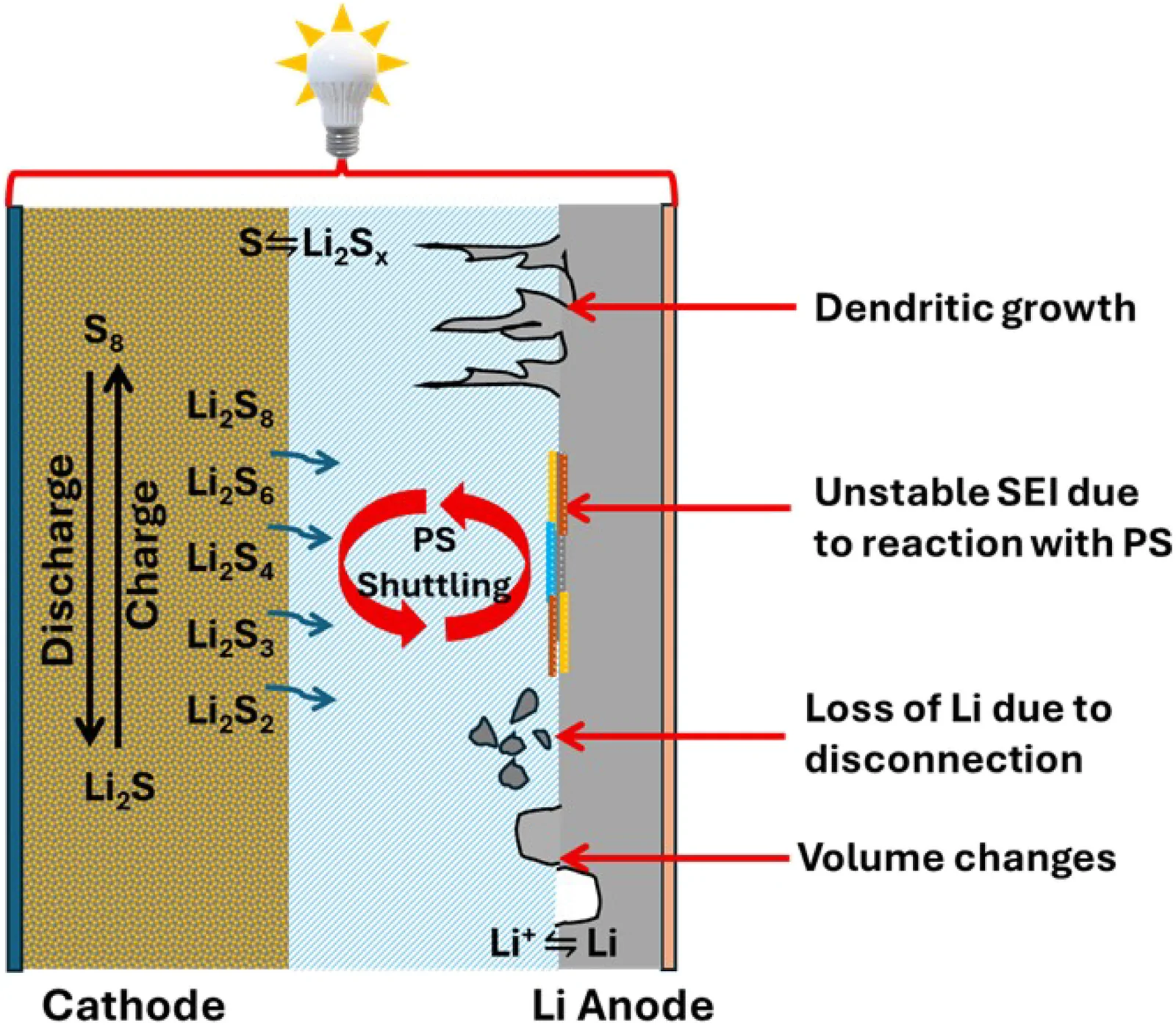
Schematic representation of working mechanism of Li–S battery, side reactions occurring at the cathode and anode electrodes during battery cycling. Dendritic growth, unstable SEI, dead Li and volume changes are the key challenges leading to unstable battery performance and premature failures.

The anode plays an important role in the performance, safety, and longevity of Li–S batteries. While S cathodes offer high theoretical energy densities, the behavior of the anode is crucial in governing processes such as Li plating/stripping, interfacial stability, and interactions with dissolved PS. Despite their high energy density, Li metal anodes present serious challenges that limit the practical implementation of Li–S batteries. Although there are multiple challenges related to the Li–S cathode, this review focuses solely on the anode, so we maintain the discussion's focus on anode-related challenges.^1,3,17^ The issues with respect to the anode include (i) uncontrolled Li dendrite growth, (ii) instability of the SEI, (iii) parasitic reactions with dissolved PS, and (iv) large volume changes during cycling (Fig. 1). Consequently, extensive research efforts have focused on stabilizing the Li metal anode and developing alternative anode architectures to improve Li–S battery cycling stability and safety.^1,8–15^

### 2.1 Challenges in anode development

• Li dendrite growth: one of the most critical issues associated with Li metal anodes is the formation of dendritic Li. Li dendrites are formed when Li deposits unevenly during repeated battery cycling. The non-uniform current distribution and uneven Li nucleation lead to the growth of needle-like dendrites. The formed dendrites can penetrate the separator and cause internal short circuits, resulting in safety risks and rapid capacity decay, Fig. 1.^8,9^

• Unstable SEI, PS shuttling and parasitic reactions: during repeated cycling, the Li metal surface continuously reacts with the electrolyte to form an SEI layer, which is unstable. In the Li–S system, this interface is particularly fragile because of the presence of soluble PS (Li_2_S_*n*_ 2 ≤ *n* ≤ 8) and its shuttling. The PS undergoes parasitic redox reactions with Li metal, leading to continuous disruption, dissolution, and reformation of the SEI. Consequently, the interphase becomes chemically and mechanically unstable, promoting uneven Li deposition and increasing interfacial resistance. This persistent degradation consumes both electrolyte and active Li, reducing coulombic efficiency and ultimately accelerating capacity fading and premature cell failure.^1,3,11,12,18–20^

• Volume change and morphological instability: Li undergoes substantial structural and morphological evolution during repeated plating and stripping processes. The continuous deposition and dissolution of Li lead to pronounced volume fluctuations, resulting in an uneven surface morphology, pore formation, and electrode roughening. These changes can cause the formation of electrically isolated “dead Li,” loss of contact with the current collector, and disruption of the SEI layer. Over prolonged cycling, such instabilities increase interfacial resistance, reduce active Li utilization and accelerate electrode degradation and ultimately leading to rapid capacity decay and poor cycling performance.^10,13,14,21^

### 2.2 Different approaches to optimize Li–S battery anodes

The development of advanced anodes is essential to fully realize the potential of Li–S batteries as next-generation high-energy-density storage systems, with benefits including higher capacity and safer operation. To overcome these limitations, researchers have focused on strategies that either stabilize Li metal or replace it with alternative materials. As illustrated in Fig. 2, various approaches have been proposed to obtain stable anodes. These include interfacial engineering through artificial SEI layers, electrolyte optimization, and anode modification. Additionally, solid-state electrolytes have been explored to further enhance the interfacial stability and suppress dendrite growth. Digital modeling and intelligent control methods have also been tried to optimize Li anodes.

**Fig. 2:**
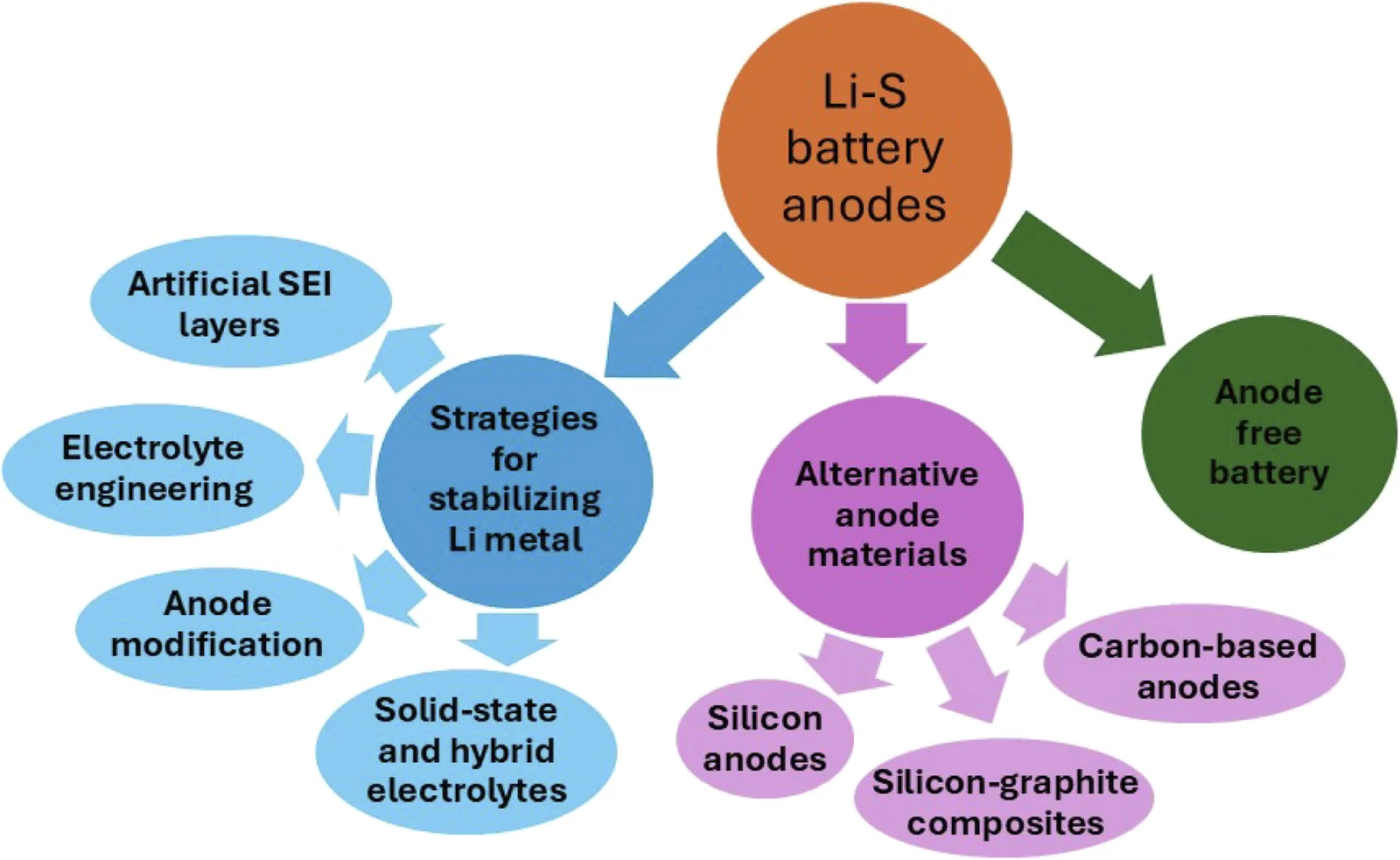
Schematic overview of the key strategies employed to design high-energy-density anodes for Li–S battery systems.

Beyond Li metal alternative anode materials such as silicon, silicon–graphite composites and carbon-based systems have been investigated to improve structural stability and safety while maintaining high energy density. Recently developments include anode-free Li–S battery configurations have attracted considerable attention because they eliminate excess Li and maximize the energy density. Overall, these strategies focus on improving Li utilization, suppressing dendrite formation, and enhancing the long-term stability and safety of Li–S battery systems.

#### 2.2.1 Strategies for stabilizing Li metal anodes for Li–S batteries

##### Artificial SEI layers

Artificial SEI layers have been extensively studied for their ability to safeguard the Li metal surface and control the movement of Li ions. The artificial SEI acts as a physical barrier while maintaining high Li-ion conductivity (Fig. 3). These protective layers can be formed using organic substances, such as hexamethylditin, and inorganic substances, such as lithium fluoride (LiF). Furthermore, polymers and hybrid organic-inorganic composites have also been the subject of research.^22–31^

**Fig. 3:**
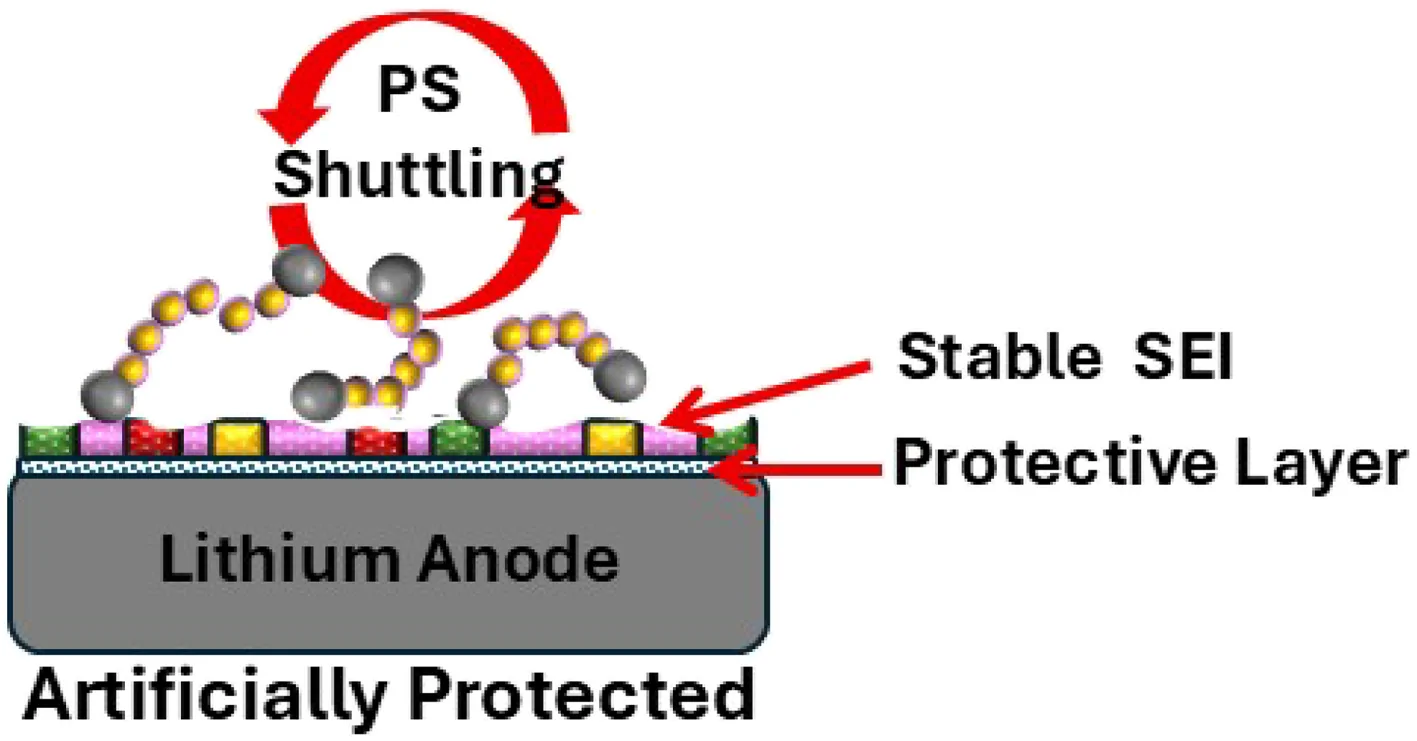
Schematic illustration of a protected Li anode with a stable artificial SEI. The artificial interphase enhances interfacial stability by regulating Li^+^ transport, suppressing dendritic Li growth and electrolyte decomposition, mitigating PS corrosion, and maintaining a robust electrode–electrolyte interface during repeated cycling.

The formation of an artificial SEI using lithium hexafluorophosphate/fluoroethylene carbonate (LiPF_6_/FEC), LiF, and sodium fluoride (NaF) has been shown to help stabilize the Li anode.^25,31^ These studies clearly demonstrate the influence of artificial SEI on the performance of Li–S batteries. Tan *et al.*^24^ proposed a simple yet effective approach to address the challenges of the Li anode by using a pretreated separator with a LiPF_6_/FEC solution. This strategy led to the formation of a LiF-rich SEI on the Li metal surface. This also generated an *in situ* gel-like blocking layer at the separator–cathode interface. The dual interfacial modification helped stabilize the Li anode and suppress undesirable side reactions during battery operation. The authors obtained stable 800 cycles with a 750 mA h g^−1^ capacity from the battery with an artificial SEI at a 0.5C rate. In contrast, batteries with no artificial SEI showed rapid capacity decay and much less capacity.^24^ Although this approach demonstrates improvements in Li metal stability and electrochemical performance, its practical applicability remains constrained by low S loading and the use of excess electrolytes, both of which deviate from commercially relevant Li–S battery configurations. Such testing conditions may overestimate the electrochemical performance and do not fully reflect the challenges associated with high energy density cells. Furthermore, the fabrication of the engineered interfacial layer often involves additional processing steps, raising concerns regarding scalability, reproducibility, and manufacturing cost.

An artificial SEI composed of LiF and NaF nanocrystals was developed using a simple solution-soaking method by Jin *et al.*^31^ The formed LiF/NaF-rich SEI layer functions as an anti-electron tunnelling barrier, effectively suppressing electron leakage from the Li surface. Both theoretical and experimental analyses demonstrated that this artificial SEI significantly reduced electron tunnelling, enabling dense and dendrite-free Li deposition beneath the protective layer even at high areal capacities (Fig. 4a and b). Compared to conventional inorganic SEI, fluoride-based LiF and NaF exhibit superior electronic insulating properties owing to their wide band gaps (9.03 and 6.2 eV, respectively). This strong electron-blocking capability effectively suppressed electron tunnelling through the SEI, thereby limiting unwanted Li nucleation within the interphase, as shown in Fig. 4c. Density functional theory (DFT) calculations were performed to determine the electronic band gaps, work functions, Li^+^ diffusion energy barriers, electron tunnelling characteristics (including critical tunnelling thickness), and interfacial energies of LiF- and NaF-based SEI components. These calculations provide theoretical insights into the electron-blocking capability, ionic conductivity, and interfacial stability of the artificial SEI. The LiF/NaF artificial SEI effectively stabilized the Li metal anode by simultaneously suppressing electron transport and facilitating rapid Li^+^ migration. The electronic conductivity of the artificial LiF/NaF SEI (9.1 × 10^−6^ S cm^−1^) was approximately one order of magnitude lower than that of the native SEI (1.37 × 10^−6^ S cm^−1^), demonstrating its enhanced electronic insulating capability. Electrical conductivity measurements and conductive atomic force microscopy showed that the LiF/NaF-rich interphase had a much lower electronic conductivity than the native SEI. This effectively blocks electron tunnelling and limiting continuous electrolyte decomposition and parasitic side reactions, Fig. 4d and e. In contrast, electrochemical impedance spectroscopy and Tafel analysis revealed enhanced Li^+^ transport kinetics, as indicated by a lower activation energy and higher exchange current density, enabling rapid ion diffusion through the artificial SEI (Fig. 4f and g). Consequently, Li deposition occurred uniformly beneath the protective interphase, producing a smooth, dendrite-free morphology, whereas bare Li showed mossy, irregular deposits (Fig. 4h). The effectiveness of the artificial SEI was further confirmed using PS-containing electrolytes. Short-circuit tests demonstrated that the protected Li anode tolerated substantially higher Li plating capacities before failure, indicating improved resistance to dendrite penetration (Fig. 4i). Microscopic analysis revealed dense, compact Li deposits beneath the LiF/NaF layer, whereas the bare Li electrode developed porous filamentous dendrites accompanied by significant volume expansion. Long-term symmetric-cell cycling with ultrathin Li anodes showed remarkably lower voltage hysteresis and stable operation over extended cycling, while bare Li experienced rapid polarization growth due to continuous SEI degradation and dead Li accumulation, Fig. 4j. Post-cycling XPS analysis further confirmed that the artificial SEI effectively mitigated parasitic reactions with dissolved PS, as evidenced by the reduced formation of S-containing decomposition products (Fig. 4k). Titration gas chromatography also revealed higher active Li retention for the protected electrode compared to bare Li (Fig. 4l). Collectively, these results demonstrate the effectiveness of a LiF/NaF-rich artificial SEI in improving interfacial stability under both conventional and practical Li–S operating conditions. The practical performance of the LiF/NaF artificial SEI was evaluated in Li–S cells using F–Li/Na and bare Li anodes. Cyclic voltammetry results showed similar redox behaviors for both systems, indicating that the artificial SEI does not hinder the reaction kinetics. However, the F–Li/Na cell exhibited a significantly reduced shuttle current and lower self-discharge (∼21% *vs.* ∼38%), confirming the effective suppression of PS-induced side reactions (Fig. 4m). In Li–S cells, at 0.5C, the protected anode delivered an initial capacity of ∼1050 mA h g^−1^ and retained ∼720 mA h g^−1^ after 500 cycles (∼68.6% retention), whereas bare Li showed rapid capacity decay, as shown in Fig. 4n. Under practical conditions (thin Li, high S loading, lean electrolyte), the bare Li cell fails within ∼30 cycles, while the F–Li/Na cell operates stably for up to 150 cycles with ∼98% coulombic efficiency, Fig. 4o. After 100 cycles, the charge–discharge profiles revealed that cells with a bare Li anode exhibited pronounced polarization and unstable voltage behavior, indicating severe anode degradation. In contrast, the cell with the LiF/NaF artificial SEI maintained more stable electrochemical characteristics (Fig. 4p). Post-cycling analysis revealed a smooth, dendrite-free surface in cells with an artificial SEI, in contrast to the severe degradation observed in bare Li. This enhanced performance is attributed to the LiF/NaF-rich SEI, which combines a strong electron-blocking capability with fast Li-ion transport, enabling uniform Li deposition and suppressing parasitic reactions, as shown in Fig. 4q.^31^

**Fig. 4:**
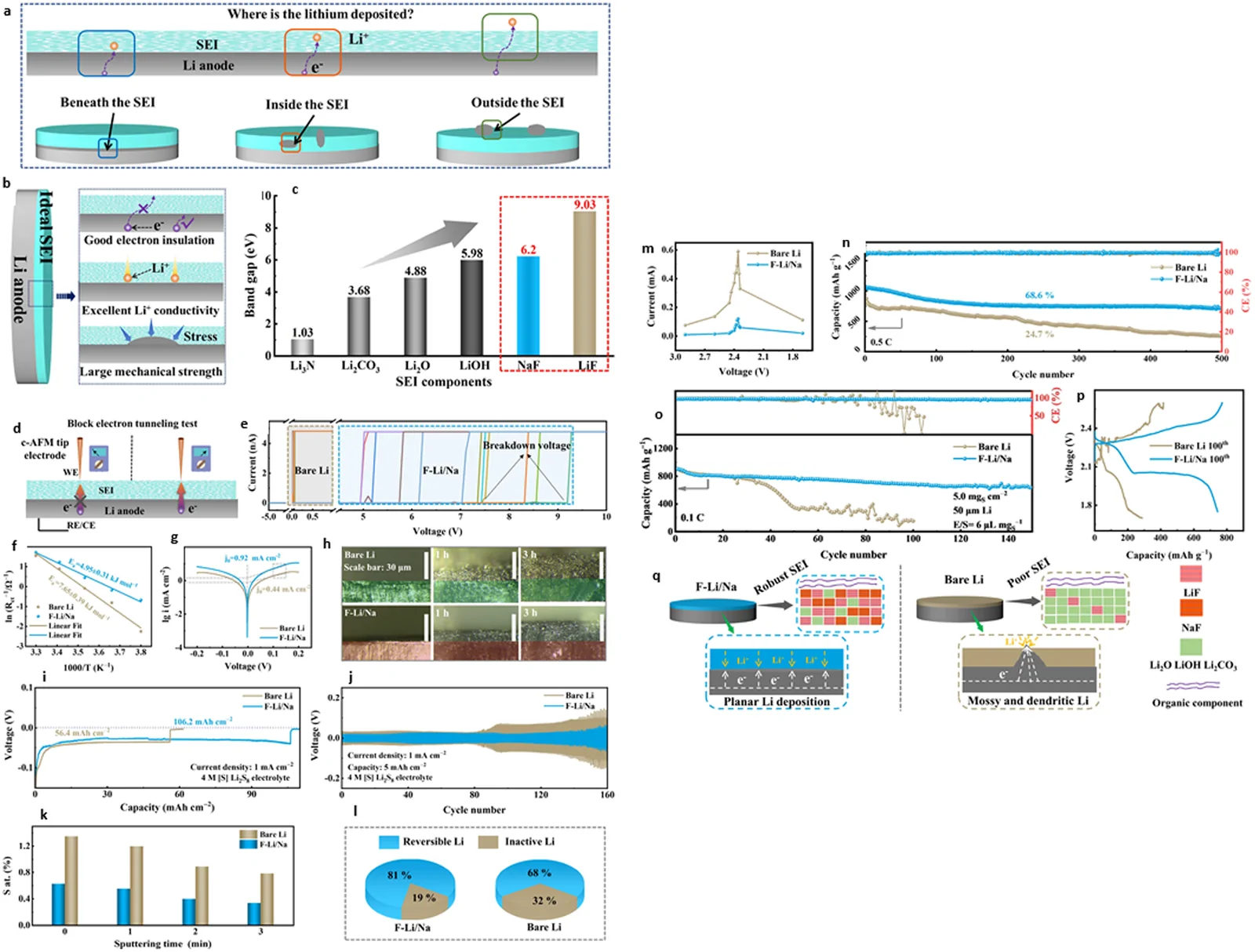
(a) Effect of SEI anti-electron tunneling ability on Li nucleation and growth. (b) Schematic of an ideal SEI. (c) Band gaps of various inorganic constituents. (d) Illustration of the nanoscale electrochemical measurement setup. (e) Current response with application of varying bias at the different regions of bare Li and F–Li/Na surfaces. (f) Activation energy for Li^+^ diffusion through the native SEI and LiF/NaF artificial SEI. (g) Tafel plots of Li‖Li symmetric cells with bare Li and F–Li/Na. (h) *In situ* optical microscopy images of bare Li and F–Li/Na at a current density of 0.5 mA cm^−2^. (i) Voltage curves at a current density of 1 mA cm^−2^ recorded during continuous Li deposition in the LiPS-based electrolyte. (j) Voltage profiles of symmetric cells with F–Li/Na and bare Li electrodes at 1 mA cm^−2^ with capacities of 5 mA h cm^−2^. (k) S elemental concentration of cycled F–Li/Na and bare Li. (l) Quantitative differentiation of the active Li content on cycled bare Li and F–Li/Na electrodes by the TGC method. (m) Shuttle current of Li–S cells as a function of voltage for the F–Li/Na and bare Li anodes. (n) Cycling performance of Li–S cells with F–Li/Na and bare Li at 0.5C. (o) Cycling performance of Li–S cells with F–Li/Na and bare Li under harsh conditions. (p) Voltage-specific capacity profiles of Li–S cells with F–Li/Na and bare Li at the 100th cycle. (q) Schematic illustration of the effect of the LiF/NaF artificial SEI on suppressing Li dendrite formation and inhibiting side reactions.^31^ Copyright 2022 by American Chemical Society.

Despite its excellent ability to suppress electron tunnelling, promote uniform Li deposition, and improve cycling stability, the LiF/NaF artificial SEI presents several challenges. The solution-soaking fabrication process introduces an additional surface modification step that may complicate large-scale manufacturing and increase the processing costs. The long-term mechanical integrity of the artificial SEI under repeated Li plating/stripping and large volume fluctuations remains to be fully established, particularly under practical operating conditions. Moreover, although the LiF/NaF layer enhances Li^+^ transport compared to the native SEI, excessively thick fluoride-rich interphases can increase interfacial resistance and adversely affect the rate capability. Finally, the long-term chemical stability and compatibility of the artificial SEI with different electrolyte formulations and high-current-density operation require further investigation before practical implementation in commercial Li–S batteries.^31^

Although artificial SEI layers provide a direct and effective means of stabilizing the Li metal surface through the formation of a protective interfacial barrier, their long-term performance is strongly influenced by the surrounding electrolyte environment. The composition and solvation structure of the electrolyte governs the formation, composition, and stability of the SEI, as well as Li^+^ transport and the extent of parasitic reactions with the dissolved PS. Consequently, electrolyte engineering has emerged as a complementary strategy that enables the *in situ* formation of robust interphases while simultaneously suppressing PS shuttling, improving Li deposition behavior, and enhancing the overall electrochemical performance of Li–S batteries.

##### Electrolyte engineering

Electrolyte engineering plays a critical role in stabilizing Li metal anodes.^32^ Li–S systems frequently utilize high-concentration electrolytes, localized high-concentration electrolytes and ether-based electrolytes with salts like lithium bis(trifluoromethanesulfonyl)imide (LiTFSI).^33,34^ The incorporation of functional additives, such as lithium nitrite (LiNO_3_), has proven especially beneficial in establishing a stable SEI on Li metal. This in turn mitigates PS reduction reactions at the anode, thus enhancing coulombic efficiency, Fig. 5.^32,35,36^

**Fig. 5:**
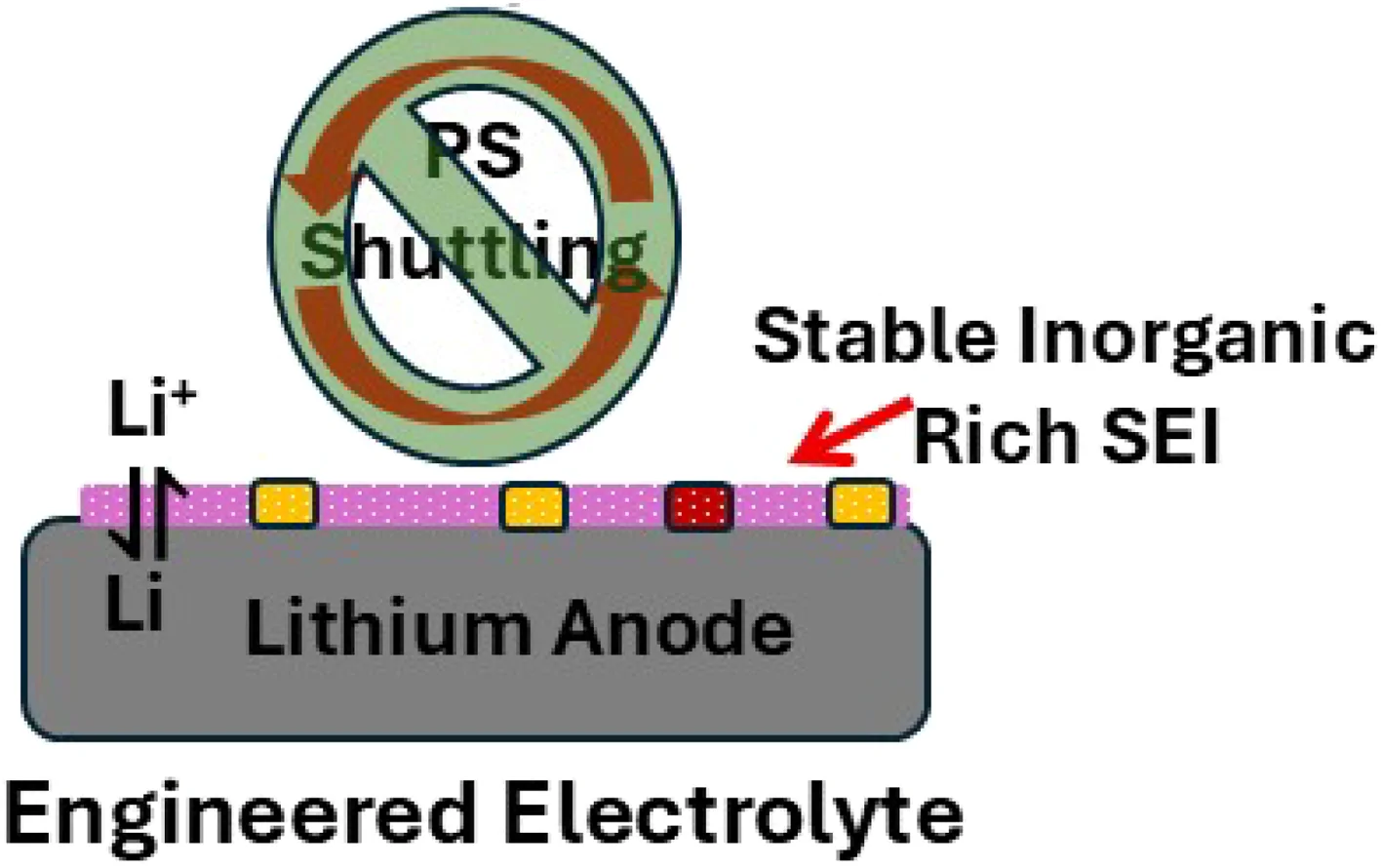
Schematic representation of Li anode behavior in the presence of engineered electrolytes. Advanced electrolyte strategies, including optimized solvents, Li salts, and functional additives, regulate interfacial chemistry, suppress parasitic reactions and PS crossover, promote uniform Li nucleation and growth, and improve the long-term stability and safety of Li–S batteries.

Liu *et al.*^33^ and Gao *et al.*^34^ reported the importance of electrolyte engineering. Liu *et al.* showed that high-concentration binary salt electrolytes suppress PS solubility and improved Li–S battery cycling stability. To address the low Li metal reversibility and PS dissolution effects, researchers have explored localized high-concentration electrolytes (LHCEs) as an electrolyte design strategy. An LHCE system with mixed Li salts, such as Li[TFSA_0.8_LiFSA_0.2_] ([TFSA] : [N(SO_2_CF_3_)_2_]), when dissolved in sulfolane and diluted with 1,1,2,2-tetrafluoroethyl 2,2,3,3-tetrafluoropropyl ether (HFE), showed positive results. This electrolyte design reduces PS solubility and suppresses shuttle reactions, thereby improving the stability of Li–S batteries. HFE, as a diluent, preserves localized high-salt coordination and improves electrolyte transport. Increased salt concentration and the use of a fluorinated diluent broaden the reduction potential gap between Li^+^ and anions, favoring controlled anion decomposition. This helps form a stable SEI with a favorable composition by improving the Li plating and stripping reversibility. Li–S pouch cells using this electrolyte showed high performance under practical conditions, including high S loading (∼5.5 mg cm^−2^) and lean electrolyte-to-S ratio (∼3 µL mg^−1^), with an energy density of approximately 280 Wh kg^−1^. These findings highlight the importance of electrolyte formulation in regulating interfacial chemistry and enhancing Li metal stability in practical Li–S battery systems. Despite its promising electrochemical performance, this strategy was evaluated under relatively idealized cell configurations and requires further validation under practical conditions with high S loading, a lean electrolyte, and a limited Li inventory. In addition, the scalability, manufacturing cost, and long-term durability of the engineered interface remain important challenges that must be addressed before commercialization.^33^

Recent studies suggest that controlling the solubility of PS in the electrolyte is a critical factor in balancing the PS shuttle effect and reaction kinetics in Li–S batteries. Electrolytes with excessively high PS solubility can aggravate the shuttle phenomenon, whereas extremely low solubility may hinder S redox kinetics. To address this trade-off, researchers have systematically investigated electrolytes with PS solubility ranging from 37 to 1100 mM (based on the S concentration). The results indicated that a solubility range of approximately 50–200 mM provided an optimal balance, as shown in Fig. 6b. This range effectively suppressed PS shuttling while maintaining favorable reaction kinetics. Based on this concept, Gao *et al.* developed a series of electrolytes termed single-solvent, single-salt, and standard salt concentration with moderate PS solubility electrolytes. These electrolytes were developed using solvents with varying degrees of fluorinated diethoxyethane (F3DEE, F4DEE, F6DEE, and F5DEE) and fluorinated-1,4-dimethoxybutane (F4DMB), and the solubility of PS in the electrolytes with these solvents was checked, as shown in Fig. 6a. To gain deeper insight, the solvation behavior of Li PS was investigated using molecular dynamics simulations. Li_2_S_6_ was chosen as a representative species to analyze both its solvation structure and cluster size distribution in 1,3-dioxolane/1,2-dimethoxyethane (DD), 1,2-diethoxyethane (DEE), and fluorinated ether solvent series (Fig. 6c and d). The presence of larger Li_2_S_6_ clusters (*e.g.*, 32- or 40-atom aggregates) indicates weaker solvent–Li PS interactions and thus poorer solvation capability. Among the solvents studied, the occurrence of larger clusters increased in the order F3DEE < F4DEE < F4DMB < F5DEE (Fig. 6e). This is consistent with the experimentally observed trend in Li PS solubility. Among these systems, Li–S cells employing fluorinated 1,2-diethoxyethane exhibited a high discharge capacity of 1160 mA h g^−1^ at 0.05C at room temperature. At elevated temperatures (60 °C), cells utilizing fluorinated 1,4-dimethoxybutane delivered an even higher capacity of approximately 1526 mA h g^−1^ at 0.05C with a coulombic efficiency of 99.89% over 150 cycles at 0.2C under lean electrolyte conditions. This electrolyte system also exhibited improved cycle life, approximately five times longer than that of conventional ether-based electrolytes, as well as excellent calendar stability, with a slight capacity recovery observed after extended rest periods. These findings highlight the strong correlation between PS solubility, solvent fluorination, and overall battery performance, emphasizing electrolyte design as an effective strategy for improving Li–S battery stability and efficiency. Although the moderate LiPS solubility electrolyte provides an effective balance between S utilization and suppression of the PS shuttle effect, several challenges remain for its practical implementation. Electrolyte formulation requires precise control of the solvation environment, which may complicate large-scale manufacturing and increase the complexity of the electrolyte formulation. In addition, the electrochemical performance was primarily demonstrated under laboratory-scale conditions. Further validation under commercially relevant parameters, such as high S loading, lean electrolyte, limited Li inventory, and high areal capacity is still required. Moreover, while moderate PS solubility improves reaction kinetics, dissolved PS are not eliminated, and parasitic reactions at the Li metal anode may still occur during prolonged cycling.^34^

**Fig. 6:**
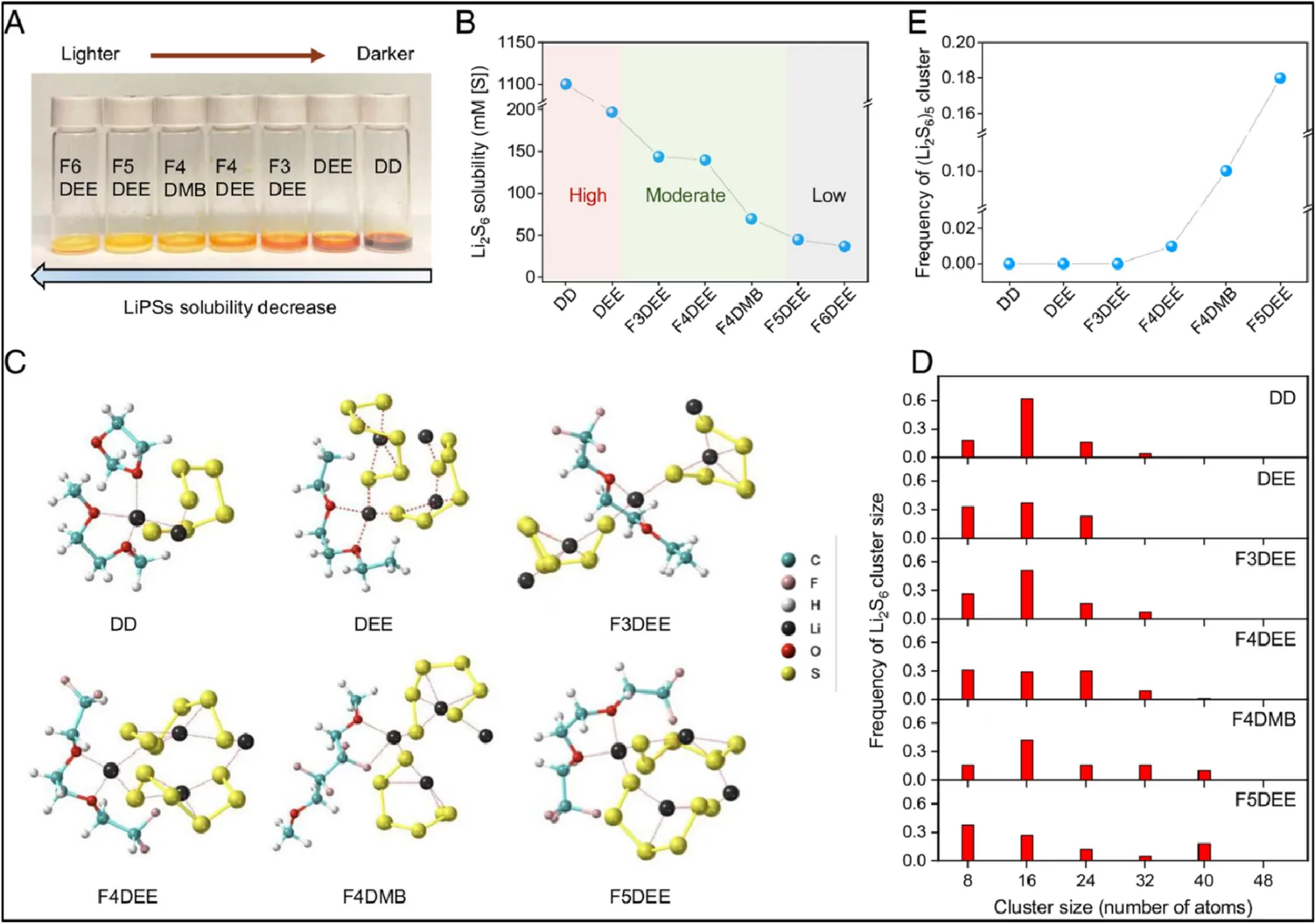
Experimental and theoretical studies on the Li PS (Li_2_S_6_) solubility and solvation structures in ether and fluorinated ether systems. (A) Photograph of 0.1 M Li_2_S_6_ (based on stoichiometric amounts of S_8_ and Li_2_S) in different electrolyte solvents. From left to right are F3DEE, F4DEE F6DEE, F5DEE, F4DMB, DEE and DD. All the solutions are saturated except for DD. (B) PS solubility of each electrolyte solvent. The solubility of Li_2_S_6_ in the designed solvents (DEE, F3DEE-F6DEE, and F4DMB) is quantified by ICP-OES measurement. (C) Representative solvation structures of the first Li_2_S_6_ solvation sheath and (D) the size distribution of Li_2_S_6_ clusters in the DD and designed solvents from classical MD simulations. (E) Frequency of the largest Li_2_S_6_ cluster.^34^ Copyright 2023 by PNAS.

Electrolyte engineering regulates Li^+^ solvation, stabilizes the SEI, and suppresses PS shuttling. However, long-term stability requires precise control over the physical processes of Li deposition. Even with optimized electrolytes, uneven Li nucleation and growth can lead to dendrite formation, morphological instability, and continuous interfacial degradation during repeated cycling. Consequently, increasing attention has been directed toward anode modification strategies, which tailor the surface chemistry and structural architecture of the anode to guide uniform Li deposition, reduce the local current density, and improve the reversibility of Li plating and stripping. These approaches complement electrolyte engineering by directly addressing the intrinsic limitations of Li metal anodes.

##### Anode modification

Different approaches have been attempted to increase the stability of metallic Li anodes. These approaches include the development of three-dimensional (3D) host materials and lithiophilic surface modification for optimized Li deposition and mitigation of parasitic reactions with PS, as shown in Fig. 7b and c. Although both lithiophilic surface modification and 3D host structures are designed to regulate Li deposition and suppress dendrite formation, they differ in their underlying mechanisms of action. Lithiophilic surface modification primarily focuses on engineering the surface chemistry of the Li metal anode or current collector by introducing lithiophilic materials that lower the Li nucleation energy barrier and promote homogeneous nucleation of Li, Fig. 7c. In contrast, 3D host structures rely on architectural design and lithiophilicity, employing porous conductive frameworks to reduce the local current density, accommodate volume changes during Li plating/stripping, and provide sufficient space for uniform Li growth. Because these strategies address complementary aspects of Li deposition, they are frequently integrated to achieve synergistic improvements in the electrochemical performance and durability of Li–S batteries. Consequently, lithiophilic modification primarily improves the initial nucleation, whereas 3D host structures govern both Li nucleation and subsequent deposition, enhancing structural stability and prolonging cycling performance. Three-dimensional (3D) conductive host materials have been investigated for controlling Li plating and reducing dendrite formation. Porous carbon frameworks, graphene scaffolds, and metal foams offer large surface areas, which lower the local current density and allow even Li deposition. In addition, these host materials can accommodate volume changes during cycling, thus improving the mechanical stability of the anode, Fig. 7b.^37–42^

**Fig. 7:**
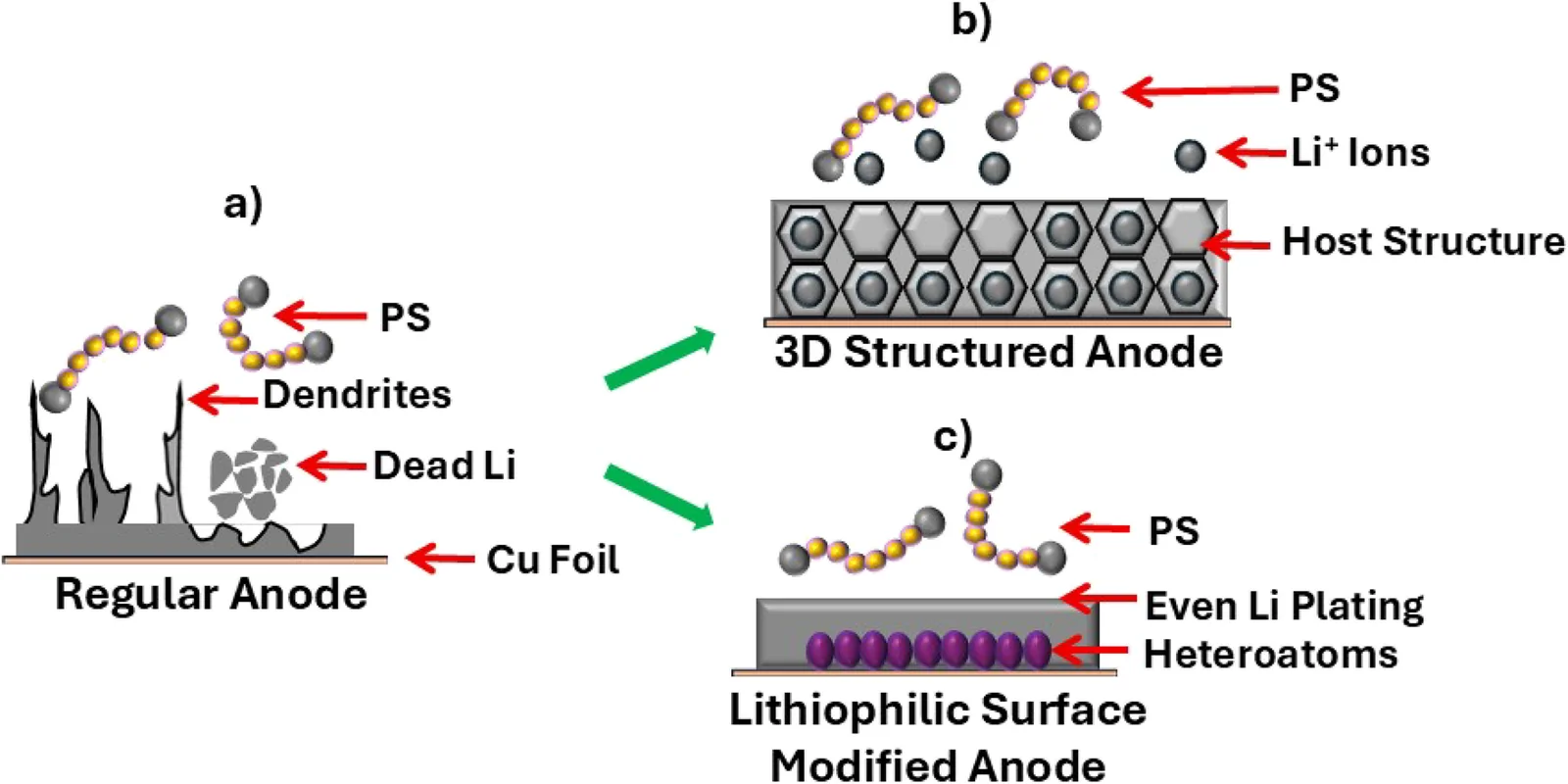
Schematic representation comparing the working mechanisms of a conventional Li anode, 3D host-structured anode, and lithiophilic surface modified anode. (a) Li anode degradation under normal circumstances. (b) In contrast, host-structured anodes provide a porous site that guides uniform Li deposition, reduce local current density and accommodates volume changes, thereby improving cycling stability. (c) Lithiophilic surface modification introduces abundant nucleation sites that lower the Li nucleation barrier, guide uniform Li deposition and regulate Li-ion distribution at the interface.

Li *et al.*^43^ used a 3D copper (Cu) foil to address the challenges associated with metallic Li anodes. This approach involved the use of a porous Cu current collector to create a 3D Cu/Li composite anode. This was achieved using a simple and easily scalable manufacturing method. The interconnected porous framework acts as a physical scaffold that confines Li deposition within the host structure while accommodating substantial volume changes associated with repeated plating and stripping. Furthermore, the substantial specific surface area inherent to the porous Cu framework diminishes the local current density, thereby promoting more uniform Li nucleation and growth relative to that of the traditional planar Cu foil. These attributes collectively enhance the interfacial stability and reduce the polarization throughout the cycling process. Electrochemical assessments employing both symmetric Li/Li cells and Li/Li_4_Ti_5_O_12_ full cells revealed improved cycling stability and prolonged cell lifespans. This underscores the efficacy of 3D host structures in stabilizing Li-metal anodes. Although the 3D porous Cu current collector effectively homogenizes Li deposition and suppresses dendrite growth by reducing the local current density, several limitations remain for its practical implementation. The fabrication of porous Cu architectures introduces additional processing complexity and may increase the manufacturing cost compared to conventional Cu foils. Furthermore, the porous framework contributes additional inactive mass and volume, which can reduce the overall gravimetric and volumetric energy densities of the battery. Although the 3D host improves Li deposition behavior, it does not fundamentally eliminate continuous electrolyte decomposition or parasitic reactions with dissolved PS, making it necessary to combine this approach with electrolyte engineering or artificial SEI strategies. Moreover, the electrochemical performance was demonstrated under laboratory conditions, and further practical validation with lean electrolyte, limited Li inventory, and pouch-cell architectures are required.^43^

Huang *et al.*^44^ showed that engineering nanostructured electrodes and modifying the surface of current collectors can effectively regulate Li plating and stripping behavior. This can mitigate dendrite growth and accommodate the significant volume changes associated with Li metal anodes. In this context, surface-modified current collectors based on Cu alloys have attracted considerable research attention. For example, a brass (Cu–Zn) mesh current collector was engineered to introduce an active surface layer through a thermally induced chemical modification process. At a current density of 1 mA cm^−2^, the Cu foil electrode exhibited a high nucleation overpotential of ∼180 mV. In comparison, the CuZn and ZnO–CuZn electrodes showed significantly lower values of ∼110 mV and ∼65 mV, respectively. Smoother voltage profiles were observed during the nucleation stage, indicating more uniform Li deposition (Fig. 8a). It also delivers a high and stable coulombic efficiency (∼97.5% over 100 cycles) along with reduced voltage polarization, demonstrating the improved reversibility of Li plating/stripping. In contrast, the Cu foil and CuZn electrodes show rapid efficiency decay and poor capacity retention owing to uneven Li deposition and continuous SEI degradation, as shown in Fig. 8b and c. This enhancement is attributed to the active ZnO active sites and the 3D conductive framework, which together promote uniform Li deposition and lower the nucleation barriers (Fig. 8d). The symmetric cell results further confirmed the superior long-term stability. After pre-depositing 3–5 mA h cm^−2^ of Li at 0.5 mA cm^−2^, the ZnO–CuZn electrode demonstrated stable cycling for up to 1200 h with minimal polarization, whereas the Cu foil showed increased polarization and voltage fluctuations after ∼300 h, as shown in Fig. 8e. Furthermore, at a higher current density of 2 mA cm^−2^, the ZnO–CuZn electrode maintained stable operation for ∼500 h, whereas the Cu foil rapidly failed due to short-circuiting within ∼60 h, as shown in Fig. 8f. This highlights its poor interfacial stability. The practical applicability of this system was tested by assembling full cells using Li/ZnO–CuZn or Li/Cu foil anodes paired with a LiNi_1/3_Co_1/3_Mn_1/3_O_2_ (NCM) cathode. The Li/ZnO–CuZn‖NCM cell demonstrated superior rate capability across current densities of 0.5–2C compared to the Li/Cu‖NCM cell (Fig. 8g). Galvanostatic charge–discharge tests (3–4.3 V) further revealed enhanced cycling stability for the modified anode, as shown in Fig. 8h and i. The Li/ZnO–CuZn‖NCM cell retained ∼94.3% of its capacity after 100 cycles, whereas the Li/Cu‖NCM counterpart showed only ∼48.5% retention. After 150 cycles, the Li/ZnO–CuZn system maintained a capacity of ∼90.2 mA h g^−1^, which was significantly higher than the ∼20.3 mA h g^−1^ observed for the Cu-based cell, as shown in Fig. 8j. These results highlight the effectiveness of the ZnO–CuZn architecture in improving the electrochemical performance and cycling durability of practical battery configurations. Although the chemically engineered ZnO-coated brass mesh effectively reduces the Li nucleation overpotential and improves Li plating/stripping stability, several challenges remain before practical application can be realized. The introduction of a 3D brass mesh inevitably increases the amount of inactive current collector material, thereby reducing the overall gravimetric and volumetric energy densities of the battery. Moreover, the study primarily evaluated the anode in Li metal and conventional full-cell configurations, whereas its performance under practical Li–S battery conditions remains unexplored. Although the ZnO layer promotes uniform Li nucleation, it does not eliminate electrolyte decomposition or PS-induced side reactions, indicating that additional electrolyte or interfacial engineering is still required. Furthermore, the long-term mechanical stability of the lithiophilic coating during repeated Li plating/stripping and its durability under high areal capacities require further investigation.^44^

**Fig. 8:**
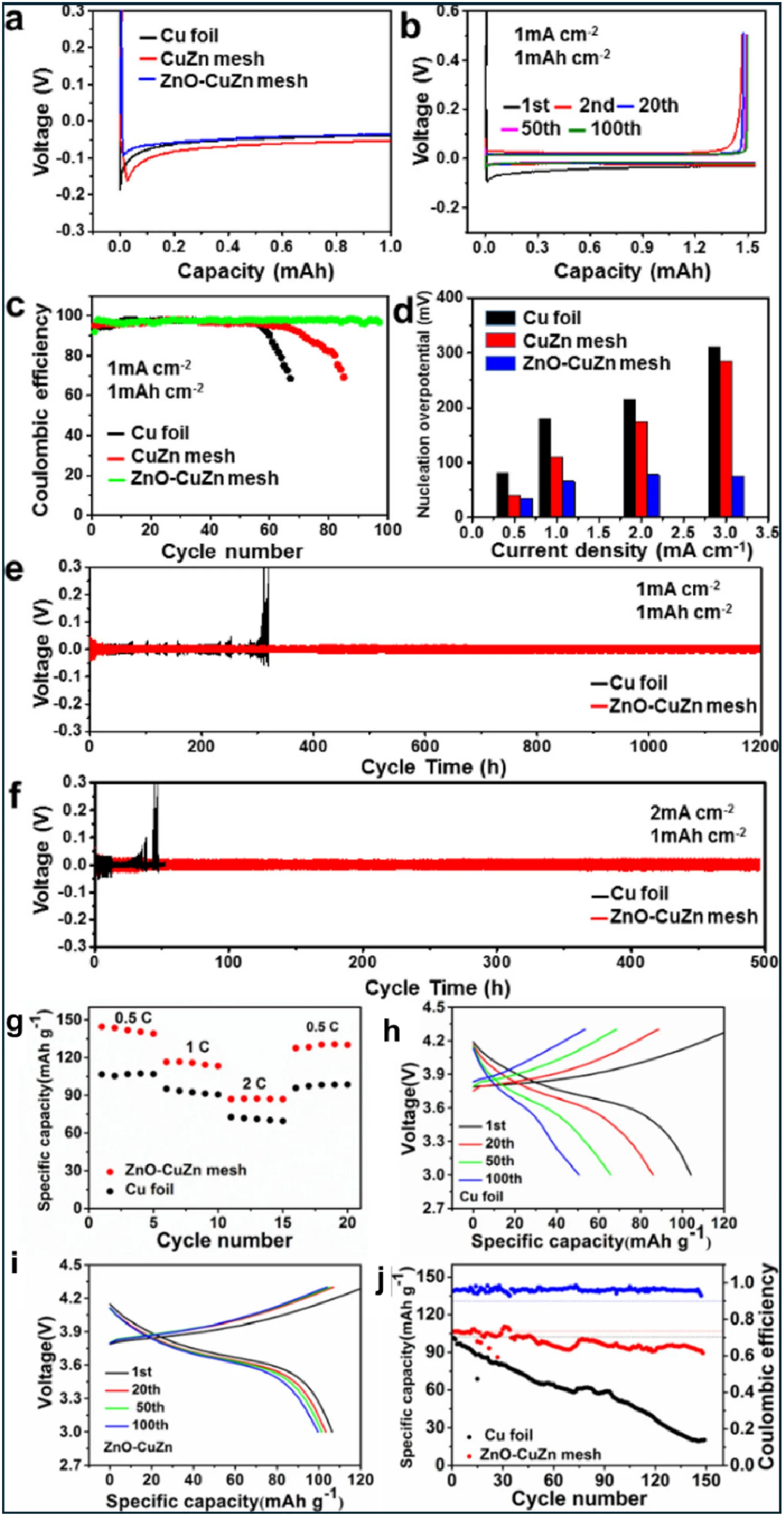
Electrochemical properties of the current collector. (a) Voltage-capacity curves of the Cu foil, CuZn mesh, and ZnO–CuZn mesh with a current density of 1 mA cm^−2^. (b) Voltage profiles of the ZnO–CuZn mesh at the 1st, 2nd, 20th, 50th, and 100th cycles with 1 mA cm^−2^ for a total of 1 mA h cm^−2^ of Li. (c) CEs of the Cu foil, CuZn and ZnO–CuZn electrodes with a cycling capacity of 1 mA h cm^−2^ at 1.0 mA cm^−2^. (d) Nucleation overpotential of the Cu foil, CuZn mesh, and ZnO–CuZn mesh at various current densities for a 1 mA h cm^−2^ of Li. Voltage–time curves of the Cu foil and ZnO–CuZn mesh in symmetrical cells with a cycling capacity of 1 mA h cm^−2^ at current densities of (e) 1 and (f) 2 mA cm^−2^. Full cell with NCM as the cathode and Cu foil and Li/ZnO–CuZn as the anode. (g) Rate performance of Li/Cu‖NCM and Li/ZnO–CuZn‖NCM cells; (h) Li/Cu‖NCM cell; (i) voltage profiles of the Li/ZnO–CuZn‖NCM cell; (j) cycling performance of the Li/Cu‖NCM and Li/ZnO–CuZn‖NCM cells at 1C.^44^ Copyright 2019 by American Chemical Society.

Liu *et al.*^45^ studied the influence of 3D conductive host structures with lithiophilicity that have been widely explored to regulate Li deposition and improve the stability of Li metal anodes. In this context, TiC/C core–shell nanowire arrays were developed as self-supported skeletons for hosting Li metal. The nanowire framework with diameters ranging from 400 to 500 nm presented several beneficial characteristics, specifically robust lithiophilicity, elevated electrical conductivity, and a porous structure. Upon the infusion of molten Li into this scaffold, an integrated TiC/C/Li composite anode was generated, in which the porous matrix served as a confined environment for Li deposition. This conductive network facilitates effective electron and ion movement simultaneously, and the internal voids provide space for Li plating and stripping during the charge–discharge cycles. Fig. 9a shows the simulation models of bare Li anodes, which show strong electric field “hotspots” at the protrusions, leading to uneven Li-ion flux, dendrite growth, and SEI instability. In contrast, TiC/C enables a uniform electric field distribution without tip effects, promoting homogeneous Li nucleation, as shown in Fig. 9b. This is attributed to the conductive nanowire network, which evenly distributes electrons and regulates Li-ion transport. Mechanistically, bare Li undergoes severe volume changes and dendritic growth, causing repeated SEI breakage and rapid electrolyte consumption (Fig. 9c). Conversely, the 3D TiC/C framework reduces the local current density, accommodates volume changes, and stabilizes the SEI, resulting in uniform Li deposition, as shown in Fig. 9d. Symmetric cell tests further confirmed this behavior: Li was reversibly stripped and deposited within the TiC/C structure without dendrite formation, maintaining a smooth and stable morphology throughout cycling, as shown in Fig. 9e–g. As a result, the structured anode demonstrated consistent electrochemical behavior characterized by minimal polarization and elevated coulombic efficiency over numerous cycles. Furthermore, when integrated with cathode materials such as LiFePO_4_ or S full cells employing the TiC/C/Li composite anode, improved capacity retention and enhanced cycling stability were exhibited, as shown in Fig. 9h and i.

**Fig. 9:**
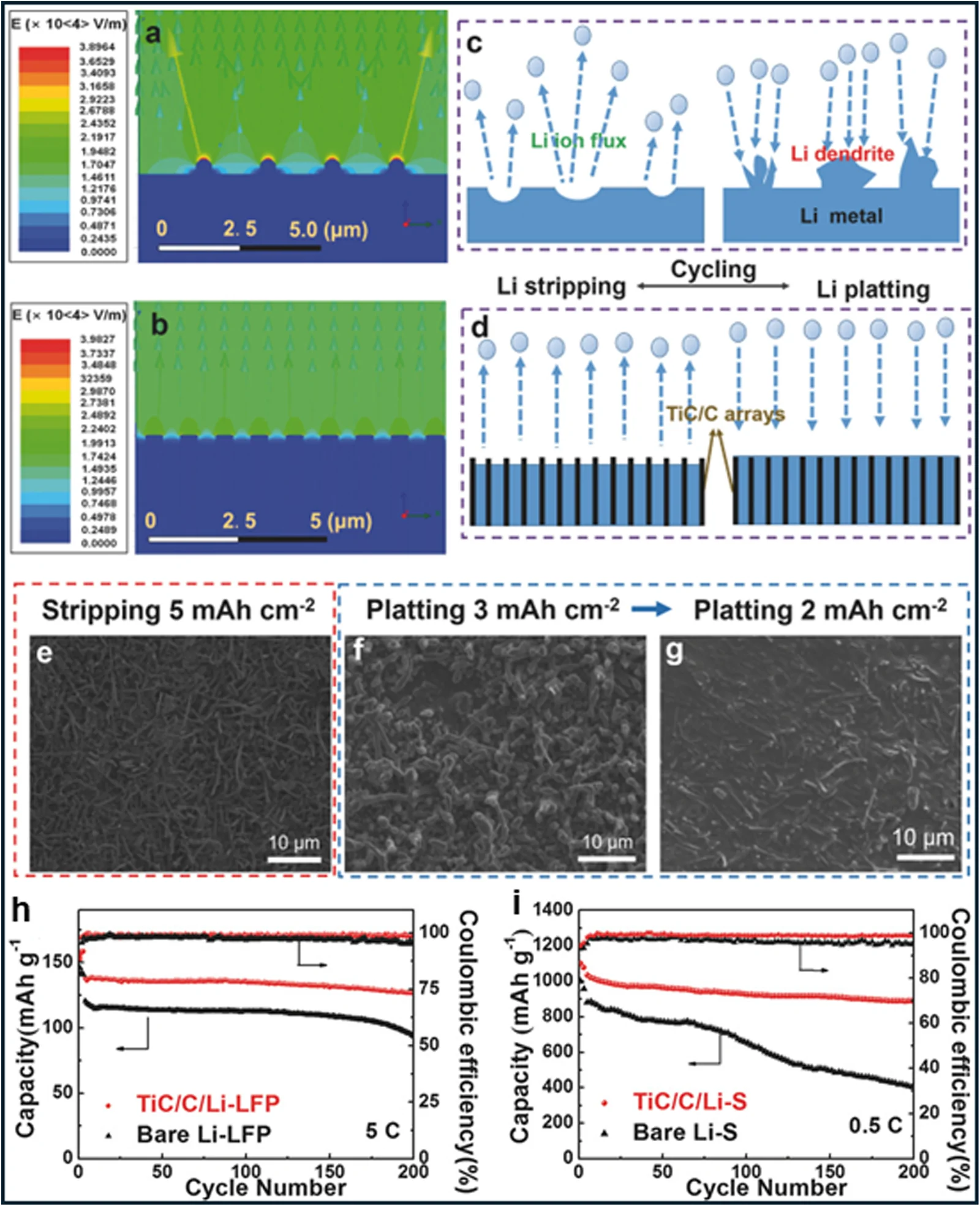
Simulation models of electric field values of (a) bare Li anode and (b) TiC/C/Li anode showing difference in the SEI stability after Li nuclei formation. Schematic diagrams of Li stripping/plating behavior of (c) bare Li metal electrode and (d) TiC/C/Li electrode showing difference in volume changes in anode. (e)–(g) Smooth and stable morphology evolution of TiC/C/Li electrode with different amounts of Li stripping/plating. (h) Capacity retention of Li–LiFePO_4_ batteries with TiC/C/Li anode showed superior performance at 5C (1C = 170 mA g^−1^). (i) Capacity retention of Li–S batteries with TiC/C/Li anode showed superior performance at 0.5C (1C = 1675 mA g^−1^).^45^ Copyright 2017 by Wiley-VCH.

Enhanced performance occurred because of the TiC/C host structure's capacity to control Li deposition, thereby decreasing the local current density, inhibiting dendrite growth, and mitigating interfacial resistance. This strategy underscores the promise of engineered conductive scaffolds for the development of stable Li metal anodes for sophisticated battery technologies. Despite its excellent ability to regulate Li deposition and buffer volume changes, the TiC/C nanowire host relies on a relatively complex fabrication process and increases the amount of inactive electrode material, potentially compromising the practical energy density. Moreover, its long-term performance under commercially relevant conditions, including high S loading, lean electrolyte, and limited Li inventory, remains to be demonstrated. Additional strategies are still required to suppress electrolyte decomposition and PS induced side reactions.^45^

An ideal host should provide sufficient internal space to accommodate the large volume changes associated with Li intercalation and de-intercalation. In addition, a high specific surface area is essential for lowering the effective current density and suppressing dendrite nucleation and growth. The host structure should also promote the formation of a robust and stable SEI that can sustain repeated interfacial and structural changes during cycling.^46^ To meet these requirements, surface modification employing lithiophilic materials has become a promising approach to facilitate uniform Li nucleation. The incorporation of lithiophilic components into host frameworks has emerged as an effective strategy for regulating Li nucleation and deposition. Materials containing heteroatom dopants such as N, ZnO or metallic species (*e.g.*, Co, Ag) can provide abundant lithiophilic sites that promote homogeneous Li nucleation and guide uniform Li growth in the electrode.^47–49^ In addition, porous carbon architectures with a high specific surface area and hierarchical pore structures enable Li deposition within the internal framework. This helps lower the local current density and suppress dendrite formation, as shown in Fig. 9b. Metal-modified conductive scaffolds further enhance the Li affinity, facilitate the efficient infusion of Li ions, and improve active Li utilization while minimizing parasitic reactions during cycling. Similarly, robust 3D ceramic-carbon composite skeletons, such as TiC/C frameworks, provide numerous nucleation sites and strong mechanical stability to accommodate volume changes during repeated plating and stripping processes. Collectively, these lithiophilic host structures enable more uniform Li deposition, improved structural stability, and significantly enhanced cycling performance of Li metal anodes. Consequently, these modifications contribute to the mitigation of dendrite formation and enhance the reversibility of Li plating and stripping processes.^50–52^

Guo *et al.*^53^ showed that lithiophilic metal layers on the Li surface regulate Li nucleation and reduce dendrite formation. This method deposits thin and heterogeneous noble metal layers, such as Ag or Au, onto Li, creating lithiophilic nucleation sites. These coatings provide numerous evenly distributed active centers for Li deposition, lowering the nucleation barrier and promoting uniform Li growth. Consequently, the Li deposits shifted from dendritic to more uniform spherical and columnar morphologies. The interfacial layer improves the stability of Li plating and stripping, leading to low polarization and better cycling durability. Symmetric cells with modified Li anodes exhibited stable cycling performance beyond 900 h with no internal short-circuiting. When paired with LiFePO_4_ cathodes, the modified anodes achieved a reversible capacity of approximately 158 mA h g^−1^ over 200 cycles and a notable rate capability of up to 5C. These results highlight the potential of lithiophilic metal coatings to enhance Li metal stability in rechargeable batteries.^53^

Although anode modification strategies, including lithiophilic surface engineering and 3D host structures, regulate Li deposition and mitigate dendrite growth, they generally rely on liquid electrolytes that remain susceptible to PS shuttling, electrolyte decomposition, and safety concerns associated with flammable solvents. To overcome these limitations, increasing research has focused on solid-state electrolytes, which offer intrinsic mechanical stability, non-flammability, and the ability to physically suppress dendrite penetration while limiting PS migration during cycling. By replacing or minimizing the liquid electrolyte, these systems provide an alternative pathway toward safer and more stable Li–S batteries with improved long-term cycling performance.

##### Solid-state and hybrid electrolytes

Solid-state and polymer electrolytes have been investigated to address the safety concerns associated with Li dendrites. The development of solid-state electrolytes has emerged as an important strategy for improving the performance and safety of Li metal anodes in recent years. Compared to conventional liquid electrolytes, solid-state electrolytes eliminate issues related to electrolyte leakage and flammability and enhance battery safety. In addition, they can effectively suppress the dissolution and migration of PS intermediates in Li–S batteries. Solid electrolytes with high mechanical moduli can suppress Li dendrite growth, and owing to less PS shuttling, the coulombic efficiency and cycling stability are also improved.^54–56^ Various solid-state electrolytes have been studied for Li–S batteries, including polymer-based electrolytes such as polyethylene oxide (PEO) with Li salts (*e.g.*, LiTFSI or LiFSI), inorganic electrolytes based on sulfide or oxide chemistries (*e.g.*, Li_2_S–P_2_S_5_ and Li_3_PO_4_), and hybrid polymer-ceramic composites. Although these methods have advantages, several challenges remain. Solid electrolytes generally have lower ionic conductivity than liquids, and the rigid solid–solid interface often causes poor contact with electrodes, hindering Li-ion transport. Introducing a small amount of liquid electrolyte can improve the interfacial compatibility and conductivity but may compromise the intrinsic safety benefits. Significant research is still needed before solid-state electrolytes can be widely used in practical Li–S batteries.^57–60^

Lyu *et al.*^60^ explored polymer-based solid electrolytes as multifunctional interfacial regulators in Li–S batteries. A conventional solid-state electrolyte composed of PEO, LiTFSI, and a porous polyethylene (PE) support was used as a reference and compared with a newly developed system incorporating a dynamic ion-selective polymer network (DSN). The DSN consists of fluorinated anionic nodes and flexible backbones with Li^+^ counterions, facilitating selective ion transport. The introduction of the DSN enabled the formation of a spontaneously phase-separated trilayer electrolyte (TLE), Fig. 10a and b. Upon casting onto the PE separator, solvent evaporation induced spontaneous phase separation, resulting in a well-defined TLE architecture with enhanced interfacial functionality and stability, as shown in Fig. 10c. This self-segregated structure produces nanometer-thick layers at both the cathode and anode interfaces, while maintaining a highly conductive bulk electrolyte matrix. In Li–S batteries, the cathode-facing layer effectively suppresses the migration of dissolved PS, thereby mitigating the shuttle effect, whereas the anode-facing layer stabilizes Li plating and stripping at the Li metal surface, as shown in Fig. 10d. These interfacial adjustments are crucially implemented without reducing ionic conductivity. *Operando* characterization and theoretical investigations suggested that dual-functional interfacial layers are essential for stabilizing Li metal behavior and enhancing PS confinement. Consequently, Li–S cells utilizing this TLE exhibited elevated initial capacities and consistent cycling performance. Furthermore, the electrolyte membrane can be produced *via* a straightforward single-step casting method, thereby underscoring its potential for scalable application in solid-state Li–S battery systems. In the presence of TLE, an initial capacity of 1369 mA h g^−1^ was obtained, and the battery showed a stable capacity for over 50 cycles at 0.2C. Although the self-segregated trilayer polymer electrolyte effectively suppresses PS migration while stabilizing the Li metal anode, several challenges remain before practical implementation. Electrochemical performance was demonstrated over a relatively limited number of cycles, and further validation under prolonged cycling, high S loading, lean electrolyte conditions, and commercially relevant areal capacities are required. In addition, maintaining a well-defined trilayer architecture and uniform phase separation over large electrode areas may become increasingly challenging during large-scale manufacturing processes. The long-term mechanical integrity of the polymer interfaces under repeated cycling, as well as their compatibility with large-format pouch cells, also requires further investigation.^60^

**Fig. 10:**
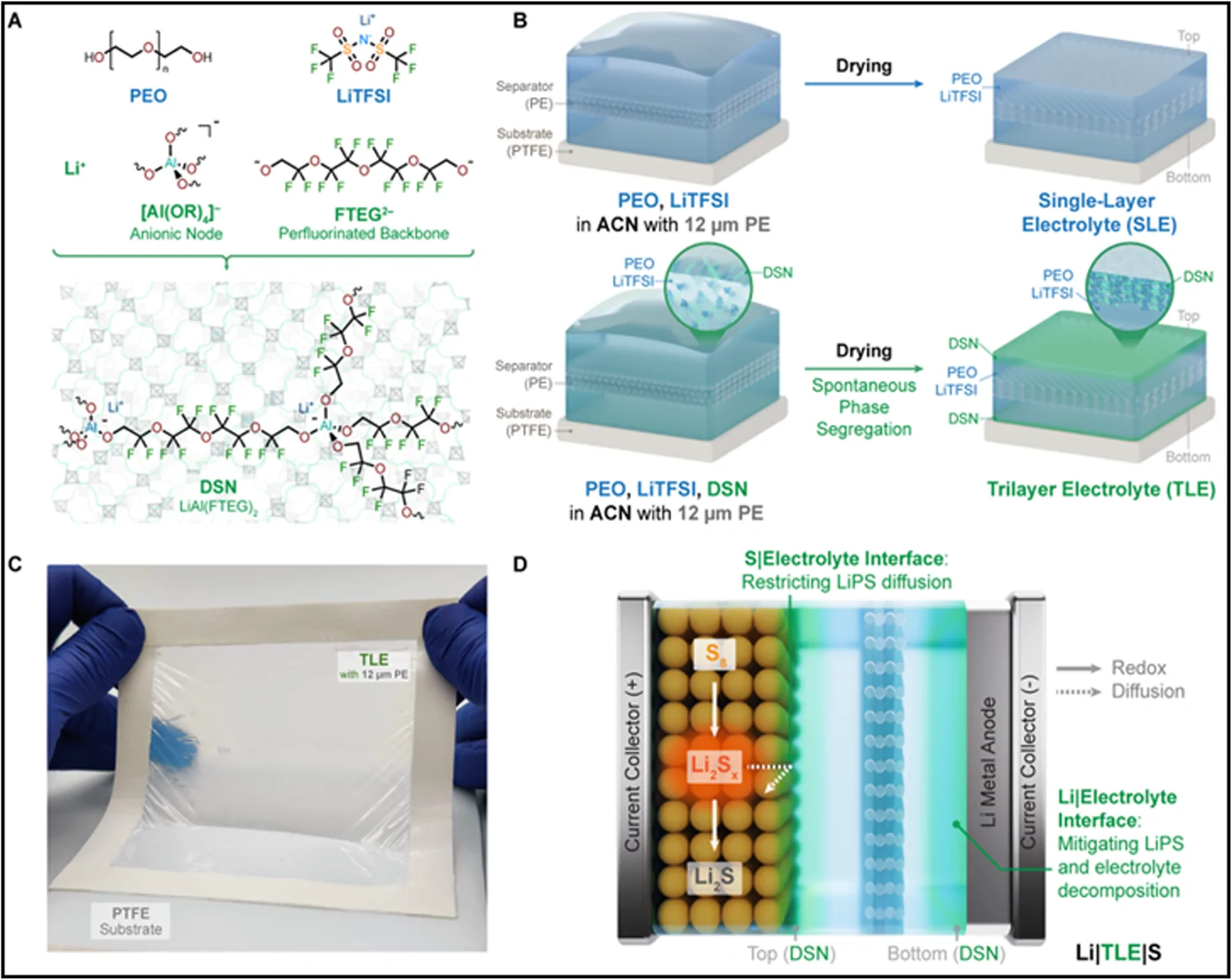
Design and preparation of freestanding self-segregated polymer solid-state electrolyte for Li–S batteries. (A) Chemical structures of electrolyte components and schematic representation of the composition of dynamic, ion-selective polymer network (DSN). (B) The preparation of single-layer polymer electrolyte (SLE) and TLE (7 wt% DSN) electrolytes through a single-step drop casting method. (C) Photo of a TLE electrolyte film of 90 mm × 90 mm size (81 cm^2^) cast in a soft tape frame. (D) Schematics of S-containing species in Li–S batteries with TLE electrolyte during discharge, and how self-segregated DSN layers mitigate the shuttling issues by interacting with PS species and stabilizing the Li metal anode. The top and bottom surfaces of SLE and TLE, when assembled into the Li–S cells, are marked corresponding to the surfaces marked in (B). The structure of DSN in panels (B) and (D) is marked by blue tetrahedra, representing [Al(OR)_4_]^−^ nodes, and green splines, representing 1*H*,1*H*,11*H*,11*H*-perfluorotetraethylene glycoxide (FTEG) backbones to illustrate its network structure.^60^ Copyright 2026 by American Chemical Society.

Interfacial instability between Li metal and garnet-type solid electrolytes remains a major obstacle to the practical implementation of solid-state Li-metal batteries. A significant challenge arises from the spontaneous formation of electronically insulating Li_2_CO_3_ and LiOH layers on the surface of garnet electrolytes (*e.g.*, Li_6.4_La_3_Zr_1.4_Ta_0.6_O_12_, LLZTO) when exposed to ambient air.^61^ These passivation layers impede Li^+^ transport, increase interfacial resistance, and promote non-uniform Li deposition, ultimately compromising the electrochemical performance. Consequently, considerable efforts have been focused on modifying or reconstructing these surface layers to facilitate fast ion transport while maintaining interfacial stability.^62,63^ Xu *et al.*^64^ to improve the interfacial compatibility between garnet-type solid electrolytes and Li metal, a mild chemical surface modification strategy was developed using magnesium trifluoromethanesulfonate (Mg(OTf)_2_). This treatment selectively converts the native, poorly conductive Li_2_CO_3_ layer on the LLZTO surface into an amorphous Li/Mg mixed-carbonate interphase enriched with LiOTf, resulting in a more ionically conductive interface. The modified composite solid electrolyte exhibited substantially reduced interfacial resistance, higher Li^+^ transference number, enhanced critical current density, and remarkable long-term stability, enabling symmetric Li cells to operate for over 5000 h and LiFePO_4_ full cells to retain 87.2% of their initial capacity after 1000 cycles. These findings demonstrate that the rational chemical reconstruction of the electrolyte surface, rather than the simple removal of surface contaminants, offers an effective route for engineering highly conductive and stable electrode–electrolyte interfaces. This work demonstrates that rational interfacial engineering of garnet-based solid electrolytes is an effective strategy for enhancing ion transport and stabilizing Li metal interfaces in solid-state batteries. Although this strategy has been demonstrated in garnet-based solid-state batteries, the underlying concept is highly relevant to solid-state Li–S batteries. Stable, ionically conductive interfaces are essential for minimizing interfacial resistance, promoting uniform Li deposition, and preventing continuous degradation. Therefore, interface engineering strategies could play an important role in enabling high-performance solid-state Li–S batteries, where efficient Li^+^ transport and stable Li metal interfaces are equally critical.^64^ Despite their promising electrochemical performance, several challenges remain the long-term chemical stability of the reconstructed interphase under high areal capacity, lean electrolyte conditions, and repeated large-volume Li deposition remains to be established. Future research should investigate the compatibility of chemically reconstructed garnet interfaces with S cathodes, evaluate their performance under practical cell configurations, and develop scalable surface modification processes that are suitable for large-scale manufacturing. Table 1 summarizes the advantages, limitations and future directions of strategies discussed to optimize the Li metal anode.

**Table 1:** Advantages and disadvantages of strategies for stabilizing Li metal anodes

Strategies	Advantages	Disadvantages	Future directions
Artificial SEI	• Provides a protective barrier that suppresses parasitic reactions between Li metal and the dissolved PS	• Mechanical instability during repeated Li plating/stripping may lead to cracking	• Develop self-healing and mechanically adaptive artificial SEI layers with high ionic conductivity
	• Regulates Li^+^ transport, promoting uniform Li deposition	• Difficult to maintain long-term interfacial stability under high current densities	• Design scalable, low-cost coating methods for practical cell manufacturing
	• Can suppress dendrite growth and improve coulombic efficiency	• Some coatings require complex fabrication techniques	• Improve long-term chemical stability under lean electrolyte and high areal capacity conditions
	• Tailorable composition using inorganic (LiF, Li_3_N, Li_3_PO_4_), polymeric or hybrid materials		
Electrolyte engineering	• Formation of a stable SEI through optimized electrolyte composition	• High-concentration electrolytes often have high viscosity and cost	• Develop low-cost electrolytes with high ionic conductivity and low viscosity
	• High-concentration or localized high-concentration electrolytes can suppress PS shuttle	• Excess salt concentration may reduce ionic conductivity	• Discover multifunctional and rechargeable additives with sustained effectiveness
	• Additives such as LiNO_3_ improve interfacial stability and reduce parasitic reactions	• Some additives are consumed during cycling, limiting long-term effectiveness	• Optimize electrolyte formulations for lean electrolyte operation and high S loading
3D host structures and lithiophilic frameworks	• Provide large surface area, reduce local current density	• Additional host materials reduce overall energy density	• Develop lightweight, highly conductive, and scalable host architectures with minimal inactive mass
	• Offers void space to accommodate Li volume expansion	• Complex fabrication processes	• Integrate multifunctional hosts that simultaneously regulate Li deposition and stabilize the SEI
	• Lithiophilic sites enable uniform Li nucleation, deposition and can significantly suppress dendrite formation	• Possible electronic or ionic transport limitations in poorly designed structures	• Evaluate performance under practical conditions, including limited Li inventory and high current densities
Solid-state electrolytes	• Non-flammable, improves battery safety	• Typically, lower ionic conductivity than liquid electrolytes	• Develop solid electrolytes with high room-temperature ionic conductivity and enhanced interfacial compatibility
	• High mechanical modulus can suppress dendrite penetration	• Poor interfacial contact between solid electrolyte and electrodes	• Engineer stable electrode–electrolyte interfaces through surface modification and composite electrolyte design
	• Can block PS migration in Li–S batteries	• High interfacial resistance can limit rate performance	• Improve processability and mechanical robustness for large-scale manufacturing of solid-state Li–S batteries

##### Digital modeling and intelligent control methods

In addition to the discussed approaches, digital modeling has provided significant insights into the interfacial instability between Li metal and electrolytes. This remains a major obstacle to the practical implementation of Li metal batteries. Multiscale computational techniques, including density functional theory (DFT), molecular dynamics (MD), phase-field modeling, finite element analysis, and computational fluid dynamics, have been widely employed to elucidate Li nucleation kinetics. Ion transport, electric field distribution, SEI evolution, and dendrite propagation across multiple length scales are also studied.^65–68^ These approaches provide mechanistic insights that are often difficult to obtain experimentally and enable the quantitative evaluation of key parameters governing Li deposition. Recently, artificial intelligence (AI), machine learning (ML), and digital twin technologies have emerged as powerful tools for analyzing large experimental and simulation datasets, predicting electrochemical performance, optimizing electrolyte compositions, and accelerating the discovery of advanced electrode materials. Despite substantial advances in artificial SEI layers, electrolyte engineering, and 3D host structures, understanding and controlling Li deposition at the electrode/electrolyte interface remains a significant challenge. Conventional experimental approaches often rely on trial-and-error optimization and post-mortem characterization, making it difficult to capture the dynamic evolution of Li nucleation, dendrite growth, and interfacial degradation processes. Consequently, increasing attention has been directed toward digital modeling and intelligent control strategies that combine physics-based simulations with data-driven approaches to predict Li deposition behavior and guide the design of materials.^69^

Digital modeling offers advantages for Li–S batteries because Li deposition is strongly influenced by PS shuttling, continuous SEI reconstruction, and the complex coupling between ion transport and interfacial reactions. Integrating *operando* characterization techniques with AI-assisted modeling could facilitate the real-time prediction of dendrite formation, dead Li accumulation, and Li inventory loss under practical operating conditions. Such predictive capabilities would enable the rational optimization of artificial interphases, lithiophilic host structures, and electrolyte formulations, thereby reducing experimental iterations and accelerating the development of high-energy, long-life Li–S batteries.^65–68^ Although digital modeling has demonstrated considerable potential, its practical application remains constrained by the limited availability of high-quality experimental datasets, insufficient integration of multiscale models, and the complexity of accurately representing coupled electrochemical, mechanical, and chemical degradation processes. Future research should focus on developing unified digital platforms that integrate *operando* characterization, multi-physics simulations, and machine learning algorithms to establish predictive frameworks for Li deposition and the failure mechanisms. Such approaches are expected to significantly accelerate the rational design of stable Li metal anodes and facilitate the commercialization of practical Li–S batteries operating under high S loading, lean electrolyte, and limited Li inventory conditions.

#### 2.2.2 Alternative anode materials for Li–S batteries

Although Li metal provides the highest theoretical capacity (3860 mA h g^−1^) and enables the exceptional energy density of Li–S batteries.^70^ The practical application of Li–S batteries has not been possible because of several critical challenges discussed in this review. To address these challenges, significant research has been conducted to explore alternative anode materials that can replace or partially substitute Li metal. Due to favorable electrochemical properties and compatibility with existing Li-ion battery technologies, silicon-based anodes, silicon–graphite composite anodes and carbon-based anodes have attracted considerable attention.^71–73^ Among other anodes, lithium titanate oxide (LTO) is widely recognized for its excellent safety characteristics and long cycle life. Although LTO anodes have undergone significant improvements, they are not considered in this review due to their low capacity in combination with S cathode.^74,75^

The radar chart provides a qualitative assessment of anode materials, including Li metal, silicon, silicon–carbon composites, and carbon (graphite), across key performance indicators, including capacity, cycling stability, safety, conductivity, and volume stability (Fig. 11). Li metal is distinguished by its remarkably high theoretical capacity and favorable conductivity. However, their application is limited by inadequate safety and volume stability stemming from dendrite formation and substantial morphological alterations during cycling. However, the use of high-capacity silicon is constrained by substantial volume expansion and diminished cycling stability. However, silicon–carbon composites demonstrate a more equilibrated performance profile, wherein the carbon matrix mitigates volume fluctuations and enhances conductivity, thereby resulting in moderate stability and safety. Despite its limited capacity graphite exhibits exceptional cycling stability, safety and structural stability thereby establishing itself as the most commercially successful anode material currently available.^70–73,76^ The preceding analysis highlights the fundamental compromises inherent in these materials, underscoring the need for judicious design methodologies to achieve an ideal balance between energy density and stability in future battery technologies.

**Fig. 11:**
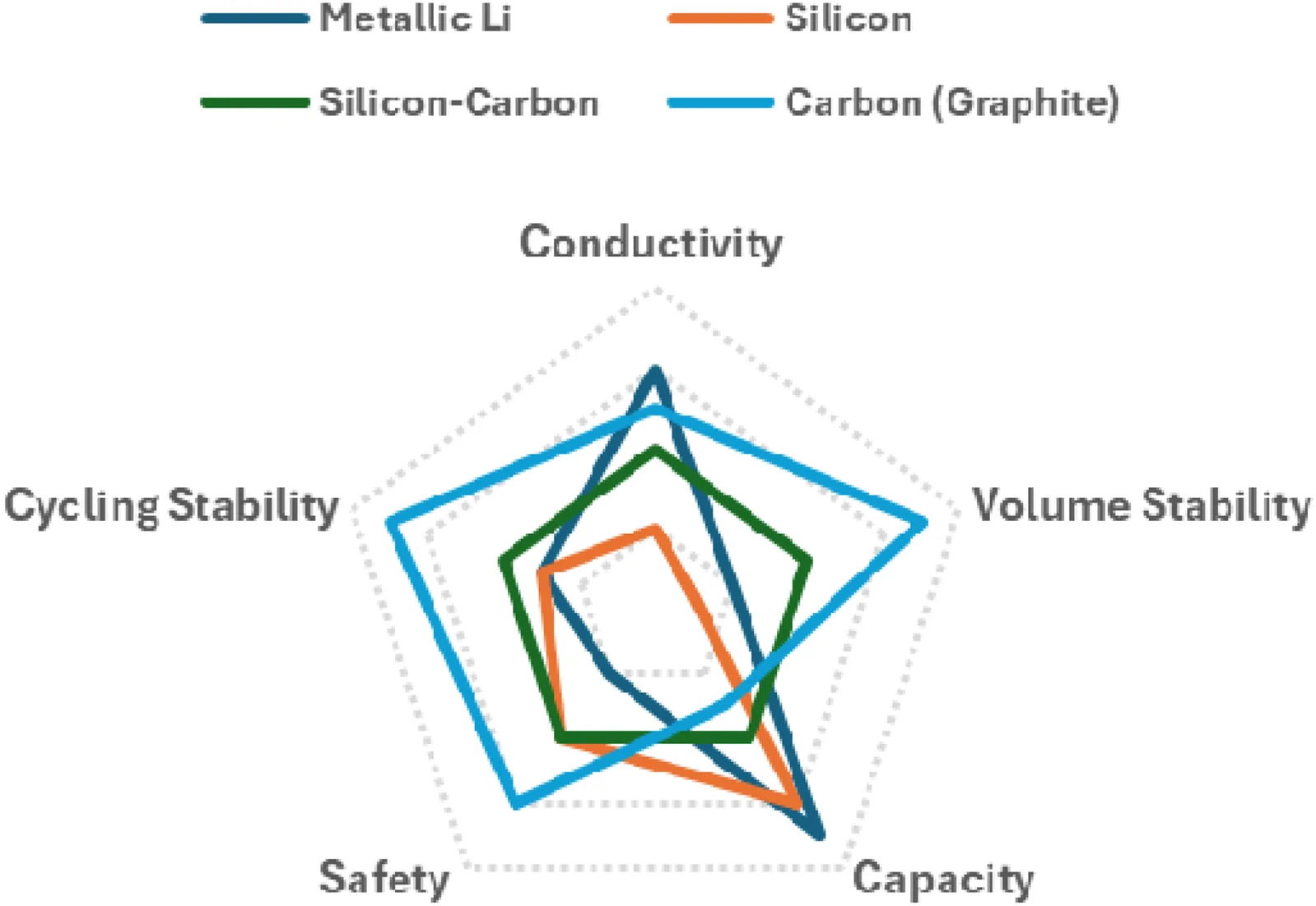
Radar chart with Li, silicon, silicon–carbon and carbon anodes compared across parameters such as capacity, stability, safety, conductivity and structural stability.

##### Silicon anodes for Li–S batteries

Silicon (Si) has emerged as an alternative anode material to metallic Li because of its exceptionally high theoretical specific capacity (up to 3579 mA h g^−1^, corresponding to the formation of Li_15_Si_4_), which is similar to that of Li metal. In addition, Si operates at a relatively low lithiation potential (∼0.4 V *vs.* Li^+^/Li), making it well-suited for high-energy battery systems such as Li–S batteries. These characteristics enable Si to retain a high energy density while potentially mitigating the safety concerns associated with Li metal batteries. However, Si-based anodes are increasingly considered as promising candidates for replacing Li metal in next-generation Li–S batteries as they offer a balance between high capacity, improved stability and enhanced safety.^76,77^

In Li–S battery configurations, Si anodes function as Li hosts rather than source. During the initial lithiation process, Li ions from the cathode react with Si to form Li–Si alloys. Subsequent charge–discharge cycles involve reversible alloying and dealloying reactions between Li and Si, respectively. The use of Si anodes can significantly reduce the safety concerns associated with Li metal batteries. Particularly, dendrite growth and short-circuiting.^78^ However, Si anodes suffer from severe volume expansion during lithiation, which can exceed 300%. This large volume change leads to mechanical stress, particle pulverization, and repeated fractures of the SEI layer. These factors result in capacity fading and poor cycling stability. In Li–S batteries these issues may be further aggravated by the presence of dissolved PS species that can react with the Si surface.^78–80^

To mitigate the obstacles in Si anodes, a range of strategies have been formulated. These include the nanostructuring of Si materials, integration of conductive carbon matrices, and design of Si–carbon composite architectures. Nanostructured Si materials, including Si nanoparticles, nanowires, and porous Si frameworks, were synthesized and tested. The discussed methods can accommodate volume expansion and improve mechanical stability during cycling. Moreover, adding Si to the conductive carbon network improves the electronic conductivity and helps Li ions move more efficiently. Improving the reaction kinetics of Si anodes remains a critical challenge for achieving high-performance Li-ion batteries.^78–83^ Cheng *et al.*^76^ proposed a contact lithiation-assisted alloying mechanism to regulate Li plating behavior and enhance interfacial reactions. This approach promotes the transformation of Li deposits from loosely distributed dendritic structures into dense, conformal layers with improved interfacial contact and Li diffusion. To elucidate the impact of Li plating on SEI stability under fast-charging conditions, detailed X-ray photoelectron spectroscopy (XPS) analyses were conducted on Si half-cell electrodes after 20 cycles (Fig. 12a and b). The electrodes were examined at 60% and 100% state of charge (SOC), representing conditions prior to and after Li plating, respectively. Significant differences in the intensities of the SEI-related species were observed between these two states for the pristine Si anode. In particular, the ratio of inorganic components (such as LiF and Li_2_CO_3_) to C–C bonds was used as a key parameter to assess changes in the SEI composition and stability (Fig. 12c). Transmission electron microscopy (TEM) was used to directly visualize the nanoscale morphology and thickness of the SEI. TEM observations revealed pronounced degradation of the SEI on the pristine Si anode at full lithiation (100% SOC), as shown in Fig. 12d and e. The initial uniform SEI layer (∼25 nm) becomes thicker (∼33 nm), irregular, and fragmented after Li plating, which increases the resistance to Li-ion transport, as shown in Fig. 12h and i. In contrast, the Si@Ag electrode formed a LiF-rich SEI with superior mechanical robustness and abundant grain boundaries, maintaining its structural integrity even at 100% SOC (Fig. 12f and g). Consequently, the modified Si-based anodes exhibited significantly improved coulombic efficiency and cycling stability compared with pristine Si. Cells with Si and Ag nanoparticles showed a very high average coulombic efficiency of 99.2% at 3C cycling rates for 300 cycles, compared with 96.4% for the pristine Si anode.

**Fig. 12:**
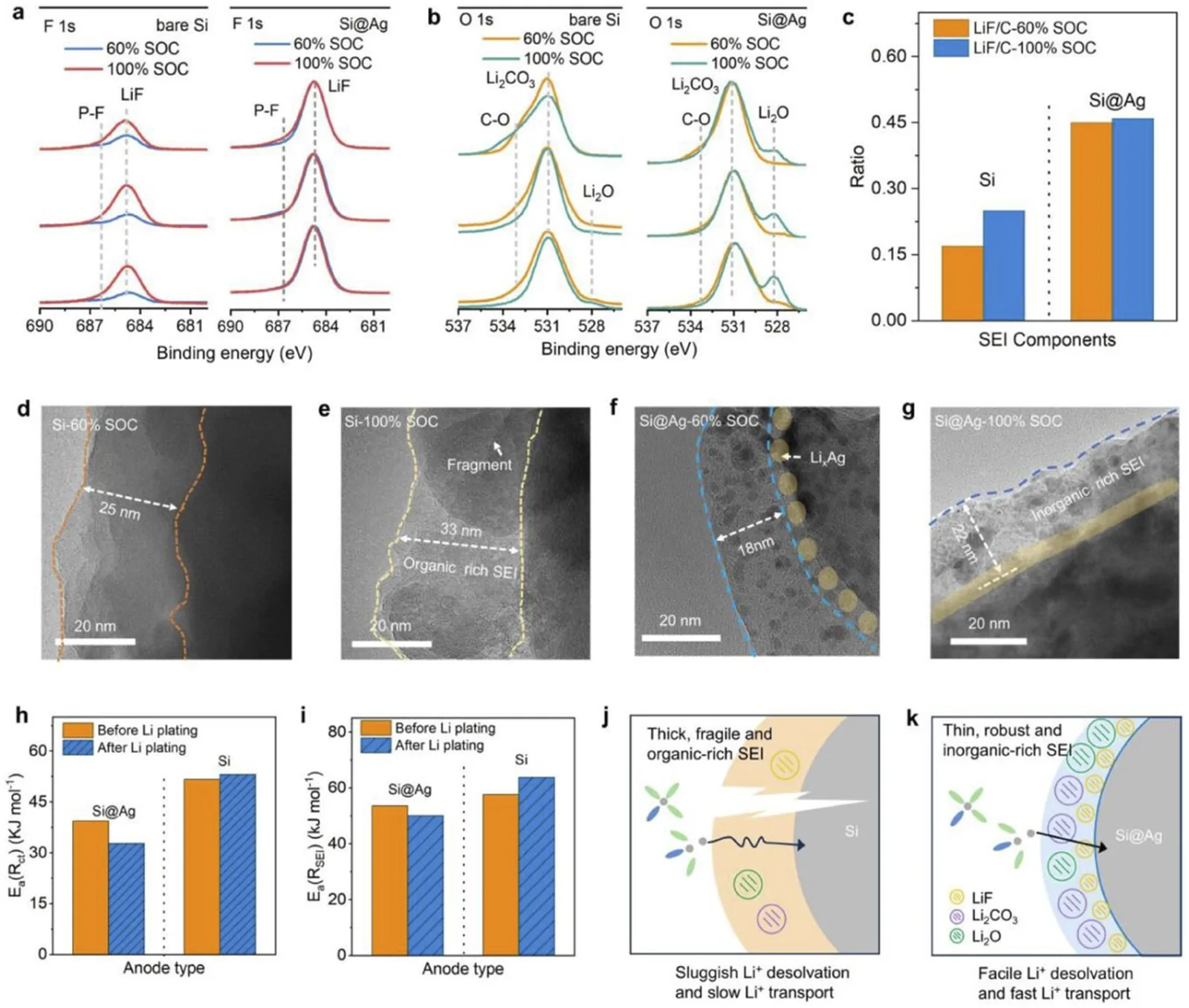
SEI investigation on anodes at 60% and 100% SOC. (a) and (b) Depth-profiled XPS spectra of F 1s and O 1s on the bare Si and Si@Ag anodes. (c) Comparison of LiF/C ratios at 60% and 100% SOC levels shows significant differences in the intensity of SEI-related species between the two states for the pristine Si anode. In particular, the ratio of inorganic components (such as LiF and Li_2_CO_3_) to C–C bonds were used as a key parameter to assess changes in the SEI composition and its stability. (d) and (e) TEM images of SEI on bare Si anode at 60% and 100% SOC shows morphology and thickness difference in the SEI. 100% SOC TEM reveal extensive degradation of the SEI on the pristine Si anode. (f) and (g) TEM images of SEI on Si@Ag anode at 60% and 100% SOC shows superior mechanical stability compared to pristine electrodes. (h) and (i) Comparisons of activation energies of *R*_ct_ and *R*_SEI_ before and after Li plating. Si@Ag showed improved activation energy and stable SEI compared to Si electrode. (j) and (k) Schematics of SEI structures on (j) bare Si SEI leads to slow Li^+^ desolvation and transportation and (k) Si@Ag anodes promotes faster Li^+^ desolvation and transportation thin and robust SEI.^76^ Copyright 2025 by Wiley-VCH.

The contact lithiation-assisted alloying strategy effectively enhances the fast-charging performance of Si anodes. However, several challenges remain in its practical implementation. This approach relies on uniformly distributed Ag nanodomains, which increases the material cost and may complicate large-scale electrode fabrication. The long-term structural stability of the Ag-modified interface under repeated volume expansion and contraction of Si also requires further investigations. In addition, although this strategy successfully suppresses Li plating during fast charging, it does not completely address the large volume changes inherent to Si anodes or the continuous evolution of the SEI during extended cycling. Finally, the electrochemical performance should be further validated under commercially relevant conditions, including high areal capacity, low N/P ratios, lean electrolyte operation, and large-format pouch cells.^76^

Electrolyte molecular engineering has emerged as an effective strategy for regulating interfacial reactions and minimizing crosstalk between electrodes in Li–S batteries. He *et al.*^83^ studied a lithiated Si–S battery configuration developed by tailoring nitrile-based electrolytes from highly solvating to weakly solvating systems. Fluoroethylene carbonate (FEC), 3-ethoxypropionitrile (EON), 1,1,2,2-tetrafluoroethyl-2,2,3,3-tetrafluoropropyl ether (TTE), and fluorinated nitrile derivatives (FnEON), where *n* is the number of moles of fluorine in the molecule, were used to optimize the electrolyte. By controlling the fluorination of ethoxy–nitrile solvents, the electronic structure and charge distribution of the solvent molecules were modified. This modification resulted in reduced PS solvation. TEM revealed that the cathode-electrolyte interphase (CEI) formed in EON/TTE–FEC was irregular and rough, which can be attributed to uncontrolled side reactions between dissolved LiPSs and the FEC additive (Fig. 13a). In contrast, the F_5_EON/TTE–FEC electrolyte produced a much thinner and more uniform CEI layer (≈10–12 nm), indicating a more stable and self-limiting interphase formation enabled by the reduced LiPS solubility (Fig. 13b). XPS further showed that this CEI is enriched in fluorine- and carbon-based species with a lower S content than that formed in EON/TTE–FEC, as shown in Fig. 13c. High-resolution F 1s and C 1s spectra confirmed a higher proportion of LiF and C–F components, which is associated with enhanced anion-derived decomposition owing to the increased participation of TFSI^−^ in Li^+^ solvation, Fig. 13d and e. On the anode side, HR-TEM analysis of the Li–Si electrodes showed that the F_5_EON/TTE–FEC electrolyte formed a uniform, compact SEI ∼14–16 nm thick. In contrast, a much thicker (≈40–50 nm) and less stable SEI developed in EON/TTE–FEC, indicating continuous electrolyte decomposition, Fig. 13f and g. Quantitative compositional analysis further revealed that the SEI formed in F_5_EON/TTE–FEC was rich in fluorinated species and contained fewer S-based components, confirming the formation of a robust anion-derived interphase and the effective suppression of parasitic reactions with LiPSs (Fig. 13h). The impedance spectra also showed a much lower resistance in F_5_EON/TTE–FEC than in EON/TTE–FEC, further confirming the influence of the engineered electrolyte (Fig. 13i). The formation of stable interfaces mitigated interfacial degradation and mechanical instability of the devices. As a result, the engineered lithiated Si–S system exhibited a high reversible capacity, excellent capacity retention over extended cycling, and high coulombic efficiency. These findings highlight the critical role of electrolyte design in decoupling electrode reactions and stabilizing interfacial chemistry, offering a promising pathway toward high-performance S-based battery systems with superior electrochemical performance. Although the fluorinated nitrile electrolyte effectively suppresses PS dissolution and stabilizes both electrodes, its electrochemical performance was demonstrated at a relatively low active material loading (2.3 mg cm^−2^), which is insufficient for practical conversion. In addition, the strategy requires further validation under practical conditions, such as lean electrolyte operation, high areal capacity, and large-format pouch cells. The cost, scalability, and manufacturability of the fluorinated nitrile solvents also require careful consideration before commercialization.^83^

**Fig. 13:**
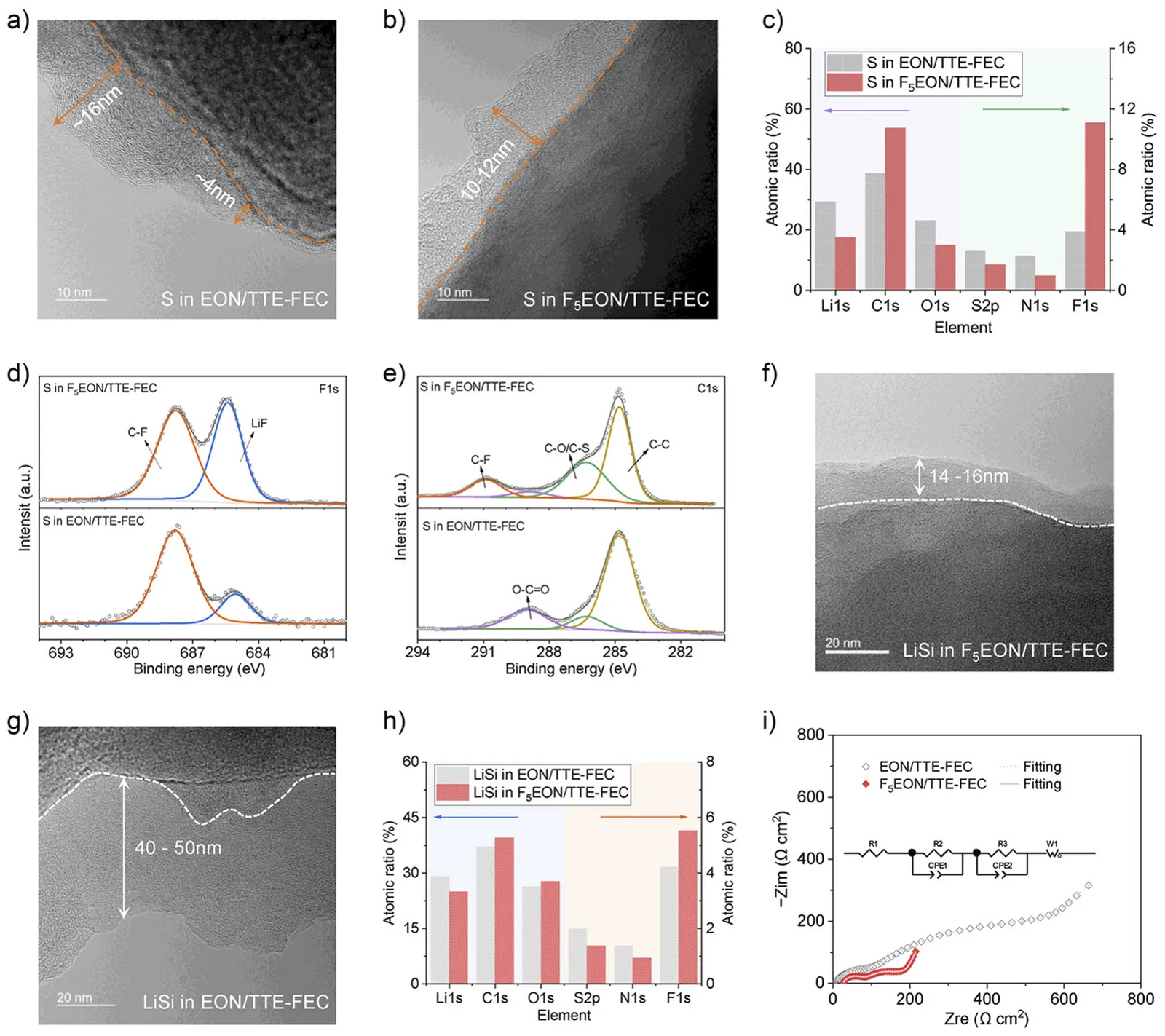
The interphases feature of the LiSi–S batteries. High-resolution transmission electron microscopy images showing the CEI formed on S cathode in (a) EON/TTE–FEC and (b) F_5_EON/TTE–FEC. (c) XPS analyses of the CEI formed on S cathode in different electrolytes after 20 cycles at 35 °C showing fluorine- and carbon-based species with a lower S content compared to that formed in EON/TTE–FEC. (d) Obtained XPS spectra of F 1s. (e) Obtained XPS spectra of C 1s. (f) High-resolution transmission electron microscopy images showing the SEI formed on LiSi anode in F_5_EON/TTE–FEC and (g) EON/TTE–FEC. F_5_EON/TTE–FEC showed uniform and lower thickness compared to EON/TTE–FEC. (h) The quantified atomic ratios on the SEI formed on LiSi anode in different electrolytes indicates, F_5_EON/TTE–FEC is rich in fluorinated species and lower S-based components. (i) The electrochemical impedance spectra of the LiSi–S cells using varied electrolytes after 20 cycles at 0.2C under 35 °C. F_5_EON/TTE–FEC electrodes showed lower impedance compared to EON/TTE–FEC.^83^ Copyright 2025 by Wiley-VCH.

Despite these advances, the practical use of Si anodes in Li–S batteries is limited owing to persistent issues such as initial Li losses, irreversible capacity fading, and compatibility with S cathodes. Addressing these challenges holds significant potential for unlocking the advantages of Si anodes for next-generation energy storage. Therefore, sustained research and innovation are crucial for realizing durable, high-performance Si-based anodes capable of enabling safer and more efficient Li–S battery systems.^80–83^ To overcome these limitations, considerable attention has been shifted toward silicon–graphite composite anodes. By combining the high capacity of Si with the structural stability, electrical conductivity, and commercial maturity of graphite, Si–graphite composites offer a balanced approach that improves the cycling stability while maintaining a high energy density.

##### Si–graphite composite anodes

Si–graphite composite anodes have attracted considerable interest as practical solutions to overcome the limitations of pure Si anodes. Graphite (G) is a well-established anode material used in commercial Li-ion batteries. Due to its excellent structural stability, good electronic conductivity, and long cycle life, it is the most preferred material. By combining Si with G, it is possible to achieve a balance between high capacity and structural stability.^84,85^ In Si–G composite anodes, G plays multiple crucial roles. G functions as a mechanically resilient matrix that mitigates the substantial volumetric expansion that occurs during Si lithiation. Furthermore, G establishes continuous electronic pathways, thereby improving the electrical conductivity of the composite electrode. G facilitates the formation of a stable SEI, which, in turn, enhances the cycling stability of the anode. The incorporation of Si into G matrices can increase the overall specific capacity of the anode compared with that of conventional G electrodes. Moreover, the presence of G helps mitigate the pulverization and structural degradation commonly observed in pure Si electrodes.^86,87^ Several methods have been developed for preparing Si–G composites. These include mechanical mixing, chemical vapor deposition, and advanced nano-structuring techniques. When integrated into Li–S battery systems, Si–G composite anodes offer improved structural stability and reduced safety risks compared to Li metal anodes. However, challenges such as irreversible Li consumption, non-compatible electrolytes and PS reactions still need to be addressed to achieve optimal performance.^88–90^

The development of scalable and safe synthesis strategies for Si-based anodes remains a key challenge for next-generation battery systems. In this context, alternative fabrication approaches have been explored to enable the controlled growth of Si nanoparticles (Si-NPs) on conductive carbon substrates. One such method was reported by Patil *et al.*^90^ who involved a two-step synthesis process that decoupled precursor decomposition from nanoparticle deposition. This allowed the *in situ* formation of Si-NPs on the nano-G frameworks. This strategy not only facilitated the uniform dispersion of Si but also improved the interfacial contact between the active material and the conductive matrix. Importantly, the use of safer precursors, such as polyvinyl alcohol or hydrogen, instead of conventional silane-based sources, reduces safety concerns and simplifies the synthesis process. Structural and electrochemical analyses indicated that the resulting Si–carbon composites exhibited homogeneous nanoparticle distribution and stable cycling behavior. Electrodes prepared using this method demonstrated good capacity retention over extended cycles, highlighting the potential of this approach for the development of high-performance and scalable Si-based anodes. Although the proposed Si nanostructure effectively improves cycling stability by mitigating particle fractures, several challenges remain. The synthesis route requires precise control over the nanostructure morphology, which may increase fabrication complexity and cost. In addition, the low tap density of nanostructured Si and large electrode surface area can lead to excessive SEI formation and reduced volumetric energy density. Further validation under practical conditions, including high areal loading, lean electrolyte, limited Li inventory, and large-format cell configurations, is also required.^90^

Interfacial engineering has emerged as an effective approach for improving the structural stability of Si@B–C nanoparticles (Si@B–C). In this context, boron oxide (B_2_O_3_) has been explored as an interfacial mediator to enhance the integration of Si and G into Si–G composite anode materials (SiG@B_2_O_3_–C). Strong interfacial bonding between Si and G was established by employing a combination of high-speed fusion and low-temperature thermal treatment. The strong bonding between Si and G leads to improved structural cohesion. Field-emission scanning electron microscopy (FESEM) was employed to examine the morphologies of pristine spherical G and SiG@B_2_O_3_–C composite at different magnifications, Fig. 14a–c. Pristine G particles exhibited a spherical morphology with an average size of ∼20 µm along with partially exfoliated surface flakes. After modification, the SiG@B_2_O_3_–C composite exhibited significant morphological evolution, where the exfoliated features were no longer visible, and the particles adopted a more uniform and well-defined spherical structure. This transformation is attributed to the high-speed fusion process, which alters the surface characteristics of the materials. Higher-magnification images further revealed that the Si nanoparticles were homogeneously distributed across the composite surface. Energy-dispersive spectroscopy (EDS) analysis confirmed the uniform dispersion of Si, along with boron and oxygen, indicating the successful formation of a stable and integrated composite architecture, as shown in Fig. 14d. The Si nanoparticle and G assembly was further confirmed from the TEM images (Fig. 14e and f). Furthermore, the application of an additional amorphous carbon coating provided enhanced electrical conductivity and mechanical integrity, resulting in a more robust composite architecture. The resulting Si–G composite anode demonstrated stable cycling performance with a high reversible capacity and good capacity retention over extended cycles (200 cycles), as shown in Fig. 14i. The overall improvements were attributed to the strengthened interfacial interactions and the protective carbon layer, which collectively mitigated mechanical degradation and maintained electrode integrity during repeated lithiation and delithiation, as shown in Fig. 14g, h and j. This strategy highlights the potential of interfacial modification using functional additives, such as B_2_O_3,_ for the development of durable and high-performance Si–G anodes. The B_2_O_3_-assisted assembly strategy effectively improves the interfacial compatibility between Si and G. Electrochemical performance was demonstrated only under relatively moderate conditions and with limited cycling. Further validation under commercially relevant conditions, including high areal loading, lean electrolytes, low N/P ratios, and full-cell configurations, is required. In addition, the long-term chemical stability of the B_2_O_3_ interfacial phase and the scalability of the high-speed fusion process remain to be established.^86^

**Fig. 14:**
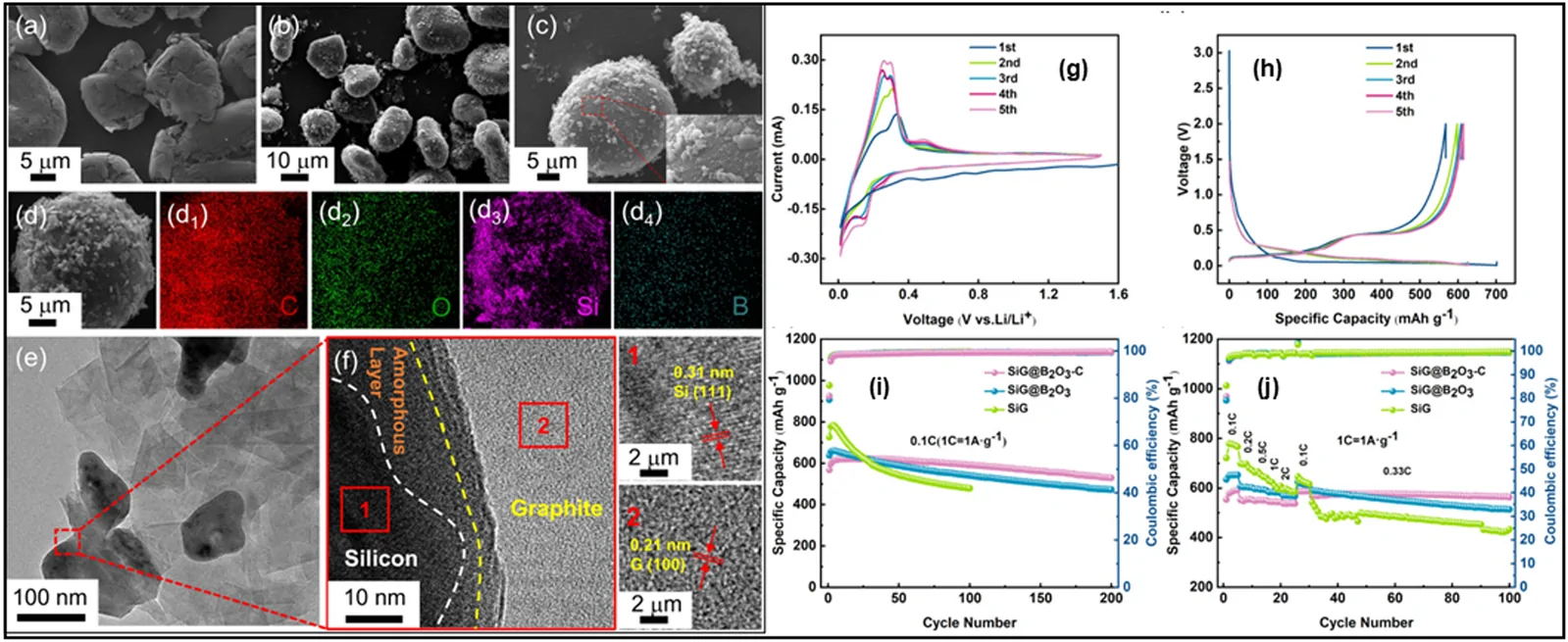
(a) FESEM image of G. (b) and (c) FESEM image of SiG@B_2_O_3_–C under different magnifications. (d) EDS element mapping of SiG@B_2_O_3_–C. (e) TEM and (f) HRTEM image of SiG@B_2_O_3_–C. (g) First five CV curves of SiG@B_2_O_3_–C at 0.1 mV s^−1^. (h) Specific capacity–voltage curves for the initial five cycles of SiG@B_2_O_3_–C at a current density of 0.1 A g^−1^. (i) Rate performance of the SiG, SiG@B_2_O_3_, and SiG@B_2_O_3_–C. (j) Cycles performance of the SiG, SiG@B_2_O_3_, and SiG@B_2_O_3_–C.^86^ Copyright 2025 by Elsevier.

Si–G composites significantly improve the mechanical stability and electrochemical performance of Si-based anodes. However, challenges such as Si volume expansion, continuous SEI evolution, and irreversible Li consumption remain. Consequently, researchers have explored carbon-based anodes as an alternative strategy. Owing to their excellent electrical conductivity, structural stability, tunable porosity, and lightweight nature, carbon materials provide robust frameworks for Li storage and transport, offering enhanced cycling stability and improved compatibility with S cathodes in Li–S batteries.

##### Carbon-based anodes

Carbon materials represent another class of alternative anodes that have been explored for use in Li–S batteries. Carbon-based anodes, including graphite (G), hard carbon, soft carbon, graphene, and carbon nanotubes, offer several advantages. This includes good electrical conductivity, structural stability, low cost and well-established manufacturing processes.^91^ G, owing to its stable layered structure, is the most common carbon anode material in Li-ion batteries. G structure allows Li to reversibly intercalate and deintercalated forming LiC_6_ with a theoretical capacity of 372 mA h g^−1^. In Li–S batteries, G anodes offer a stable and safe alternative to Li metal, thus avoiding the risk of dendrite formation.^92^ Hard and soft carbon materials have also been studied as possible anode materials. Hard carbon has a disordered structure and nanoporous design and can store Li through a combination of intercalation and pore-filling. This unique storage method allows hard carbon to provide higher capacities than G, while also maintaining good cycling stability.^93,94^ Graphene and carbon nanotubes, which have advanced carbon nanostructures, have been studied as potential anode materials. This is because of their excellent electrical conductivity and large surface area. These materials improve electronic conductivity and improve Li-ion diffusion, resulting in enhanced overall electrochemical performance of Li–S batteries.^95,96^ However, carbon-based anodes generally demonstrate increased capacity fading relative to Li-metal or Si anodes, a characteristic that could potentially diminish the overall energy density of Li–S battery systems. Furthermore, carbon materials may also react with soluble PS thereby compromising long-term cycling stability.^97^

To address these issues, different approaches have been attempted. Jeschull *et al.*^98^ reported the use of polyacrylic acid sodium salt (PAA–Na) as a protective electrode binder that facilitates efficient and reversible Li intercalation into G within ether-based electrolytes. This advancement allowed for the development of a stable “lithium-ion-sulfur” cell, which incorporated a lithiated G negative electrode and an S positive electrode utilizing the common 1,3-dioxolane : 1,2-dimethoxyethane solvent system that aligns with the electrochemistry of Li–S batteries. G–S Li-ion cells achieved average coulombic efficiencies of approximately 99.5%, in contrast to less than 95% for traditional Li–S cells, and exhibited significantly improved capacity retention, considering cell balancing factors. This high efficiency is attributed to the enhanced interfacial stability of the G electrode, which effectively suppresses the PS redox shuttle and self-discharge. The lithiated graphite anode effectively reduces the PS shuttle and self-discharge, and its relatively low specific capacity and higher operating potential compared with Li metal inevitably decrease the overall energy density of Li–S batteries. In addition, the study was conducted under relatively low S loading and low C-rate (C/10) conditions, which do not fully represent the practical operating conditions. Further validation under high S loading, lean electrolyte, and long-term cycling is required to assess its commercial viability.^98^

In their study, Huang *et al.*^99^ introduced a novel Li–S battery design that employed a hybrid anode composed of electrically connected G and Li metal to manage unwanted surface reactions on Li. The lithiated G positioned in front of the Li metal anode acted as an artificial, self-regulating solid-electrolyte-interface layer. This layer actively managed the electrochemical reactions and reduced harmful side reactions, resulting in notable performance enhancements, as shown in Fig. 15a. Li–S cells with these hybrid anodes achieved a high specific capacity of ∼900 mA h g^−1^ at a current density of 1370 mA g^−1^ (≈0.8C). Even at a much higher rate of 13 790 mA g^−1^ (≈8C), a capacity exceeding 450 mA h g^−1^ was retained, highlighting the excellent rate capability. Moreover, the system exhibited significantly improved capacity retention upon prolonged cycling. Fig. 15b and c present the long-term cycling performance and coulombic efficiency of Li–S cells with the hybrid anode at different current densities, demonstrating superior stability across all rates. At 612 mA g^−1^ the capacity stabilizes after the initial cycles and maintains ∼850 mA h g^−1^ over 200 cycles with minimal initial loss and 99.5% coulombic efficiency throughout cycling, as shown in Fig. 15b. Similar enhancements were observed at higher current densities and extended cycling durations, underscoring the critical role of the hybrid anode in improving durability. Notably, at 1737 mA g^−1^, the cell retained ∼800 mA h g^−1^ after 400 cycles, corresponding to a capacity decay of only ∼11% and a coulombic efficiency of ∼99% (Fig. 15c). Despite the significant improvement in interfacial stability, hybrid anodes increase the electrode complexity and the content of inactive materials, potentially compromising the gravimetric energy density of the battery. The strategy also continues to rely on LiNO_3_ electrolyte additive and metallic Li, indicating that electrolyte decomposition and Li consumption are mitigated rather than eliminated. In addition, its performance under commercially relevant conditions, configurations remain to be demonstrated. Table 2 summarizes the advantages, limitations and future directions with resepct to the alternative anode materials.^99^

**Fig. 15:**
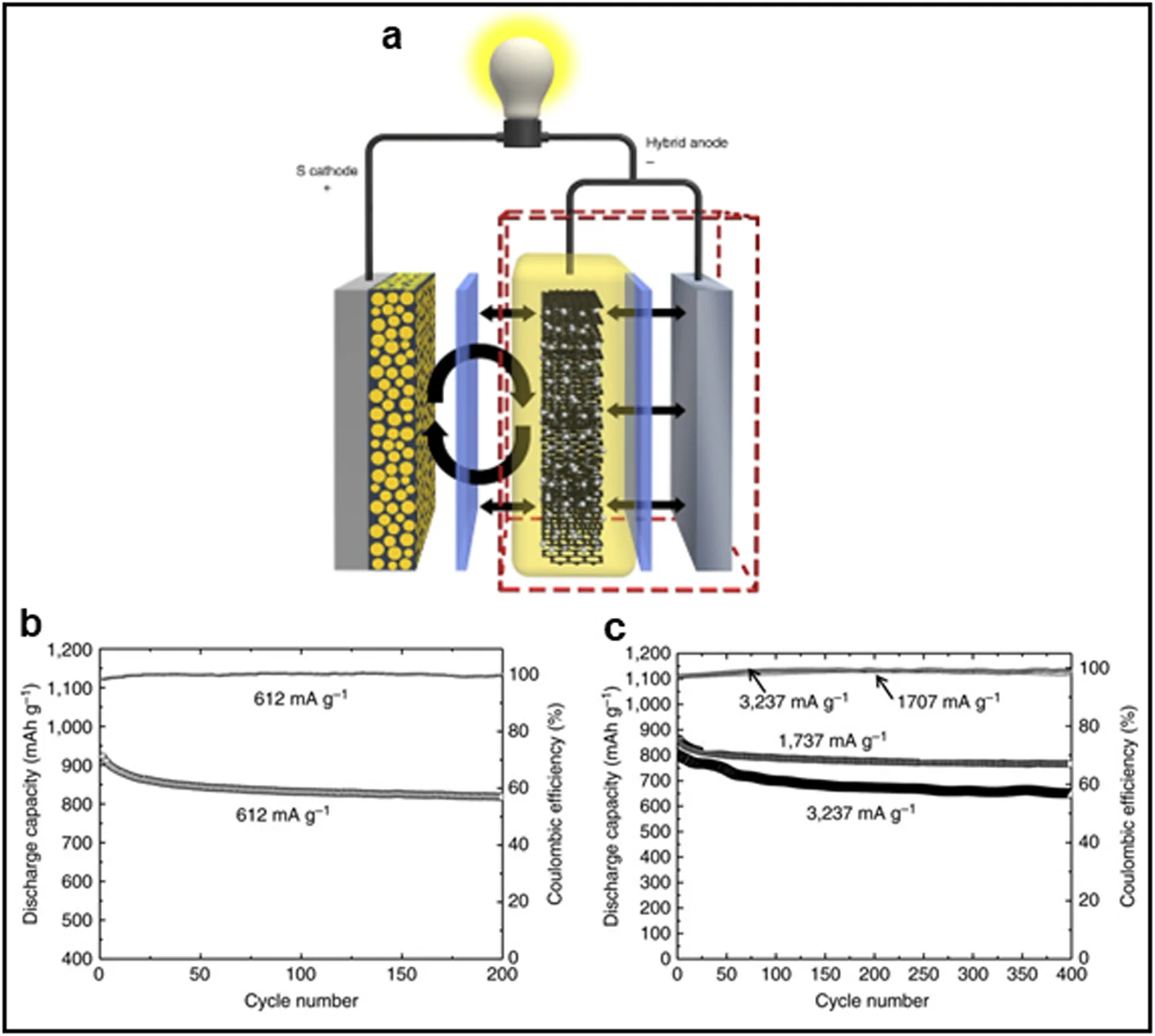
(a) Schematic of the hybrid anode design to manipulate the surface reactions on Li–S batteries. (b) and (c) Long-term cycling behavior and corresponding coulombic efficiencies of hybrid Li–S cells at different rates.^99^ Copyright 2014 by Springer Nature.

**Table 2:** Advantages and limitations of alternative Li–S battery anode materials

Material	Advantages	Disadvantages	Future directions
Si anode	• Very high theoretical capacity (∼10× G)	• Severe volume expansion (∼300%)	• Develop nanostructured and porous Si architectures to accommodate volume changes
	• Abundant and low-cost	• Poor cycling stability	• Engineer stable SEI layers and advanced binders to improve cycling stability
		• Low intrinsic conductivity	• Enhance conductivity through composite design and evaluate performance under practical conditions, including high areal loading and lean electrolyte operation
Si-carbon composites	• Buffered volume expansion	• Still suffers gradual degradation	• Optimize Si/carbon composition and microstructure to balance energy density and durability
	• Improved conductivity	• Complex synthesis and higher cost	• Develop scalable and cost-effective synthesis routes
	• Better cycling stability than pure Si	• Lower capacity than pure Si	• Investigate long-term stability under practical operating conditions and integrate multifunctional binders and electrolyte additives to further enhance interface stability
	• More balanced performance		
Carbon-based anodes	• Excellent cycling stability	• Low theoretical capacity	• Design advanced carbon architectures with higher Li-storage capability through heteroatom doping and porous nanostructures
	• High safety and structural integrity	• Limited energy density	• Develop hybrid carbon-based composites to increase energy density while maintaining structural stability
	• Mature, commercially viable technology	• Not suitable for next-gen high-energy systems alone	• Explore their application in anode-free and Li–S battery systems to improve interfacial stability and suppress PS crossover

Although extensive efforts have substantially improved the stability of conventional Li–S anodes, the reliance on Li metal or pre-lithiated anode materials continues to limit the practical implementation of Li–S batteries because of irreversible Li loss, dendrite formation, and safety concerns. Moreover, the use of excess Li lowers the cell-level energy density and increases the material cost. These limitations have prompted growing interest in anode-free Li–S batteries, which have a fundamentally different cell configuration in which Li is stored exclusively in the cathode and electrochemically deposited onto a bare current collector during the initial charging process. By eliminating excess Li, this strategy has the potential to maximize the specific energy and simplify cell design, although it also imposes stringent requirements on Li utilization efficiency, interfacial stability, and electrolyte compatibility.

#### 2.2.3 Anode-free Li–S batteries

Anode-free Li–S batteries have recently emerged as a promising strategy for maximizing the energy density of Li–S systems while simplifying the cell architecture. In contrast to conventional Li–S batteries that employ excess Li metal as the anode, anode-free configurations utilize a bare current collector, typically Cu or carbon-coated Cu, without pre-deposited Li. In this configuration, the Li required for cell operation originates from the S (Li_2_S) cathode and is plated onto the current collector during the first charging process. This design eliminates the need for excess Li metal and significantly increases the gravimetric and volumetric energy densities of batteries. This approach reduces safety risks associated with metallic Li, Fig. 16.^100–102^ Despite these advantages, the implementation of anode-free Li–S batteries presents several critical challenges. The absence of excess Li means that irreversible Li loss during cycling cannot be compensated, making the system highly sensitive to parasitic reactions. The PS shuttle effect can lead to severe Li consumption and low coulombic efficiency. Moreover, Li plating on a bare current collector often occurs non-uniformly, resulting in dendritic growth, formation of electrically isolated “dead Li” and rapid capacity degradation. The unstable SEI formed during repeated cycling further increases Li loss and reduces cycle life.^100,103^

**Fig. 16:**
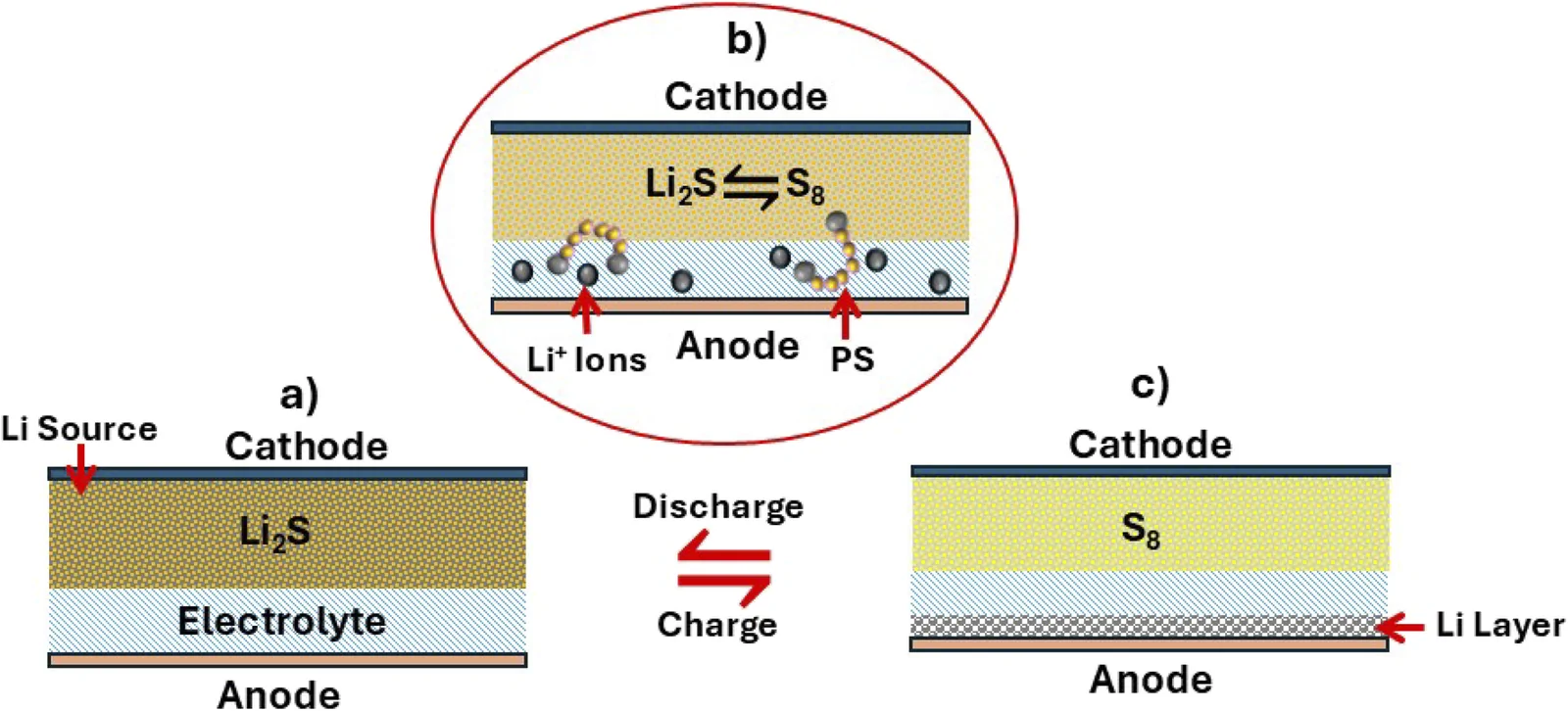
Schematic illustration of the working mechanism of anode-free Li–S battery. In this configuration (a) Li is initially stored in the cathode as Li_2_S and no excess Li metal is present at the anode side. (b) During the first discharge Li^+^ ions are released from the cathode and (c) gets plated onto a bare current collector (CC) (*e.g.*, Cu) forming a Li metal layer *in situ*. Upon charging the deposited Li is stripped and returns to the cathode, enabling reversible cycling.

To address these issues, various strategies have been proposed to stabilize Li deposition and improve the reversibility of Li cycling in anode-free systems. For instance, the surface modification of current collectors with lithiophilic materials, such as metals, metal oxides, or heteroatom-doped carbon coatings, have shown to promote uniform Li nucleation and deposition. Furthermore, electrolyte engineering, including the use of high-concentration electrolytes, localized high-concentration electrolytes, and functional additives, can help form stable interphases and suppress parasitic reactions. Finally, structural design of 3D current collectors and incorporation of protective interfacial layers have also been explored to regulate Li deposition behavior and improve cycling stability.^101,103–105^

A deep understanding of the lithiation/delithiation process in materials is crucial for advancing Li–S batteries. Nanda *et al.*^101^ presented a groundbreaking full-cell design using a Li_2_S cathode and a bare Cu foil as the anode. By removing excess Li, they achieved a truly Li-limited Li–S battery. This system operates through the reversible plating and stripping of Li on a host less substrate, specifically a copper foil. Battery performance strongly depends on the efficiency of Li deposition. The Li_2_S full cell showed impressive capacity retention, maintaining a coulombic efficiency of 96% over 100 cycles. This marks a significant improvement over a similar Li-plating-based full cell with lithium iron phosphate (LiFePO_4_) cathodes. The durability of the system was linked to the stabilization of Li deposition. This was aided by PS intermediates that formed protective Li_2_S and Li_2_S_2_ layers on the deposited Li. Removing the metallic Li anode improved both the energy density and safety of the battery. The stable and effective performance of Li plating-based Li_2_S full cells highlights their importance in Li–S battery research and the development of reversible Li anodes. The 3D conductive framework effectively regulates Li deposition and improves cycling stability, the strategy does not eliminate irreversible Li loss or dead Li formation during repeated cycling. In addition, the electrochemical performance was demonstrated under relatively mild conditions, and further validation under practical operating conditions, including high areal capacity, lean electrolytes, and long-term cycling, is required. The additional fabrication steps associated with the 3D host may also increase manufacturing complexity and cost.^101^

The design of lithiophilic 3D current collectors has emerged as a promising approach for stabilizing Li deposition in anode-free battery systems. In this regard, Cheng *et al.*^105^ used a silver-modified 3D copper framework created through thermal evaporation to apply a consistent Ag lithiophilic coating. The porous 3D framework provided a large surface area, abundant Li nucleation sites, and reduced local current density, whereas the lithiophilic Ag coating lowers the Li nucleation barrier and facilitated homogeneous Li growth. Compared with planar Cu collectors, the combined effect of the Ag layer and 3D architecture substantially decreased the Li deposition overpotential and promoted uniform Li deposition throughout the internal channels of the scaffold rather than on the outer surface. *Ex situ* SEM observations further confirmed that the Li deposited on Ag@3D-Cu was denser, more compact, and significantly less prone to dendritic growth. The engineered current collector exhibited markedly improved electrochemical performance in anode-free Li_2_S-based cells. At a Li_2_S loading of 3.8 mg cm^−2^, the Ag@3D-Cu‖Li_2_S cell delivered an initial discharge capacity of 752.4 mA h g^−1^ and retained 424.1 mA h g^−1^ after 180 cycles, outperforming the unmodified 3D-Cu collector. The initial coulombic efficiency increased from 65.3% to 70.7% and rapidly stabilized at approximately 97% after several cycles, indicating highly reversible Li plating/stripping behavior. In addition, the cell maintained a good rate capability from 0.05 to 1C and exhibited a recoverable capacity upon returning to a low current density. Notably, the architecture remained effective even at an ultrahigh Li_2_S loading of 14.6 mg cm^−2^, achieving an initial areal capacity of 7.4 mA h cm^−2^, which exceeds the areal capacity of typical commercial Li-ion batteries.^105^

The underlying concept is highly relevant to practical Li–S batteries because Li deposition is governed by both the electrolyte chemistry and the nucleation characteristics of the current collector. The Ag coating provides energetically favorable lithiophilic sites that reduce the nucleation overpotential, while the 3D porous framework distributes the current more uniformly and accommodates deposited Li within its internal channels. This combination suppresses dendrite formation, minimizes “dead Li” accumulation, and improves the reversibility of Li plating/stripping under limited Li conditions, which is particularly important for anode-free Li–S batteries. Despite their promising electrochemical performance, several challenges remain before this strategy can be applied to practical anode-free Li–S batteries. This study employed Ag coating, which introduces additional material costs, and the thermal evaporation process may present scalability challenges for large-area manufacturing. Although high Li_2_S loadings were demonstrated, the cycling stability at the highest loading remained limited (44.1% retention after 40 cycles), indicating that Li inventory loss and interfacial degradation have not yet been fully resolved. Furthermore, the cell relied on a modified separator to suppress PS shuttling, suggesting that stable operation still requires the simultaneous optimization of the cathode, electrolyte, separator, and current collector.^105^

Even after significant advances in recent years, anode-free Li–S batteries continue to face several critical challenges that limit their practical applications. The most fundamental issue is the continuous loss of the active Li inventory during cycling. In addition to irreversible Li consumption through parasitic reactions with dissolved PS and repeated SEI formation, non-uniform Li nucleation and growth on the Cu current collector resulting in electrically isolated “dead Li” during stripping. The accumulation of dead Li, together with ongoing SEI growth, progressively depletes the limited Li inventory available in anode-free cells, leading to rapid capacity decay and a shortened cycle life. These degradation pathways become even more pronounced under practical conditions employing lean electrolytes, high S loading, and high areal capacities, where insufficient electrolyte and limited Li inventory accelerate interfacial degradation, increase the local current density, and reduce Li plating/stripping reversibility. Consequently, achieving a high coulombic efficiency (>99.9%), minimizing irreversible Li loss, and maintaining stable Li deposition over extended cycling remain critical requirements for practical anode-free Li–S batteries. Future research should therefore focus on integrated strategies combining robust interfacial engineering, electrolyte optimization, lithiophilic current collector design, and controlled Li nucleation to mitigate discussed challenges.^100,106–110^Table 3 summarizes the advantages, limitations and future directions of anode free approach.

**Table 3:** Advantages and limitations of anode-free Li–S batteries

Advantages	Disadvantages	Future directions
• Eliminating excess Li metal significantly increases gravimetric and volumetric energy density	• Even minor irreversible Li loss during cycling leads to rapid capacity fading	• Develop high-Coulombic-efficiency electrolytes and multifunctional electrolyte additives to minimize irreversible Li loss
• Reduces risks associated with Li dendrite growth and short-circuiting	• Continuous Li consumption and poor reversibility of Li plating/stripping shorten lifespan	• Engineer lithiophilic current collectors and artificial interphases to promote uniform Li nucleation and suppress dead Li formation
• Absence of a pre-deposited Li anode enables a thinner and lighter cell configuration	• Li plating on bare current collectors can be non-uniform leading to dendrite formation	• Improve S cathodes to reduce PS crossover and preserve Li inventory
• Li is supplied solely from the cathode promoting efficient use of active materials	• Dissolved PS can react with plated Li and accelerate degradation	• Optimize cell designs under practical conditions, including lean electrolyte, high S loading, and low N/P ratios
• Eliminates the need for Li metal foil and reduces material and processing costs	• Requires highly optimized electrolytes and interfacial engineering for stable operation	• Validate long-term performance in large-format pouch cells while developing scalable and cost-effective manufacturing strategies

Table 4 summarizes the electrochemical performance of Li–S batteries with different anode designs and stabilization approaches discussed in this review. It includes the initial discharge capacity, capacity retained after prolonged cycling, and the corresponding C-rates used for the evaluation. To ensure a fair and meaningful comparison, only studies demonstrating long-term cycling stability, strong performance at moderate-to-high C-rates, and operation under practically relevant conditions (such as realistic S loading and electrolyte content) were included.

**Table 4:** Provides a summary of the electrochemical performance achieved from approaches considered to stabilize anodes in Li–S batteries^a^

Anode type	Measured C-rate	1st/2nd cycle capacity (mA h g^−1^)	Obtained cycles	% capacity retention	Reference numbers
Artificial SEI	0.5	1050	800	66.7	24
	0.5	1050	500	71.4	31
Electrolyte engineering	0.5	900	400	55.6	36
	0.2	1100	140	45.5	34
3D host structures and lithiophilic frameworks	4.0	109.6 (Li-ion)	500	80	43
	0.5	1100	200	86	45
Solid-state electrolytes	0.2	500	50	96.0	60
	1.0	139 (Li-ion)	1000	87.3	64
Si (with S cathode)	0.2	925	400	57.3	83
	0.06	600	50	95.8	110
Si–G composites (Li-ion battery)	0.2	∼500	100	99.0	90
	0.1	575	200	92.0	86
Carbon-based anodes	0.1	∼1000	120	91.4	98
	0.8	∼875	400	80.0	99
Anode-free	0.2	∼600	140	50.0	101
	0.1	∼752	180	56.4	105

a Direct comparisons of anode performance across reported studies should be approached with caution due to variations in current density, cycling protocols, and cell configurations. Accordingly, Table 4 is intended to highlight general performance trends rather than provide a rigorous quantitative ranking.

## 3. Conclusion

In summary, the performance, safety, and practical application of Li–S batteries are intrinsically linked to the stability of the anode. Li metal, while offering unmatched theoretical capacity and enabling high energy density suffers from critical challenges such as dendrite growth, unstable SEI and parasitic reactions with soluble PS. Therefore, addressing these issues is essential for the realization of durable and high-performance Li–S systems. This review provides an overview of the different approaches used to address the challenges of Li anodes in Li–S batteries.

This review highlights a range of effective strategies developed to stabilize Li metal anodes. Approaches such as artificial SEI layers and electrolyte engineering have demonstrated significant improvements in interfacial stability and suppression of side reactions. The use of 3D host structures and lithiophilic surface modifications have enabled more uniform Li deposition by mitigating dendrite formation and accommodating volume changes. In parallel, solid-state and hybrid electrolyte systems offer promising pathways for enhancing safety while maintaining electrochemical performance. The exploration of alternative anode materials such as Si, Si–G composites, carbon-based options, and anode-free Li–S systems are discussed in detail. These alternatives reveal promising strategies for enhancing structural stability and reducing dendrite-related risks while providing a reasonably high capacity. However, these alternatives often have their own limitations, emphasizing the importance of thorough optimization at both the material and system levels.

Looking forward, the development of advanced Li–S batteries will require integrated strategies that simultaneously address interfacial stability, electrode compatibility, and electrolyte design. Emphasis should be placed on scalable material synthesis, long-term cycling stability, and realistic operating conditions. Ultimately, rational anode engineering combined with a holistic understanding of cathode–anode interactions will be key to unlocking the full potential of Li–S batteries for next-generation energy storage applications.^111–114^

## 4. Future perspectives

Although considerable progress has been made in advancing Li–S batteries through the development of improved cathodes, anodes, electrolytes, and separators, several fundamental challenges still hinder their practical commercialization.^115–119^ Future research should focus on simultaneously achieving high energy density, long-term cycling stability, fast-charging capability, enhanced safety, and cost-effective manufacturing. In particular, the instability of the Li metal anode remains one of the most critical barriers. Because no single strategy can address all the inherent challenges, the development of synergistic approaches that can integrate advanced material design, electrolyte engineering, and interfacial regulation is essential for realizing practical, high-performance Li–S batteries.

For Li metal anodes, the development of robust and adaptive artificial SEIs capable of accommodating repeated volume changes while maintaining high ionic conductivity and chemical stability remains a priority in the field. A deeper mechanistic understanding of Li nucleation, dendrite evolution, dead Li formation, and interfacial degradation under practical conditions, including high areal capacities, lean electrolytes, and limited Li inventories, is also required. The combination of *operando* characterization with multiscale modeling and AI-assisted data analysis can provide valuable insights into these processes and accelerate the rational design of next-generation anodes. Electrolyte engineering will continue to play a central role in the development of localized high-concentration electrolytes, functional additives, and polymer or solid-state electrolytes that regulate Li^+^ solvation, suppress PS shuttling, and promote stable inorganic-rich SEI formation. Likewise, advanced lithiophilic host structures with lightweight, porous, and mechanically robust architectures should be designed to enable uniform Li deposition while remaining compatible with scalable and cost-effective manufacturing processes. Alternative anodes, such as Si, Si–carbon composites, and advanced carbon materials offer attractive pathways for improving safety while maintaining competitive energy densities.

Anode-free Li–S batteries are one of the most promising approaches for maximizing the energy density by eliminating excess Li metal. However, their practical realization is still limited by irreversible Li inventory loss, dead Li formation, low coulombic efficiency, and unstable Li plating. Future efforts should focus on the integrated optimization of lithiophilic current collectors, artificial interphases, S cathodes, and electrolyte formulations to achieve highly reversible Li cycling.

Finally, bridging the gap between laboratory demonstrations and commercial Li–S batteries require standardized testing under practical conditions, including high S loading, lean electrolyte operation, low N/P ratios, high areal capacity, and pouch-cell configurations. A holistic approach combining advanced materials design, interface engineering, electrolyte optimization, and realistic cell evaluation is crucial for translating recent scientific advances into commercially viable, high-energy-density Li–S batteries.

## Author contributions

Anjana A conducted the literature survey, prepared the figures and tables, and contributed to the writing and revision of the manuscript. Manu U. M. Patel conceived and supervised the review, developed the overall structure and direction of the manuscript, wrote the initial draft, coordinated the writing process, and finalized the manuscript.

## Conflicts of interest

The author declares no known competing financial interests or personal relationships that could have influenced the work reported in this paper.

## Data Availability

This is a review article and does not report any new experimental or computational data. All data supporting the findings of this study were derived from previously published articles, which have been appropriately cited in the reference list. Any additional information related to this review can be obtained from the corresponding author upon reasonable request.
